# Species richness, cultural importance, and prioritization of wild spices for conservation in the Sudano-Guinean zone of Benin (West Africa)

**DOI:** 10.1186/s13002-018-0267-y

**Published:** 2018-11-15

**Authors:** Konoutan Médard Kafoutchoni, Rodrigue Idohou, Anthony Egeru, Kolawolé Valère Salako, Clément Agbangla, Aristide Cossi Adomou, Achille Ephrem Assogbadjo

**Affiliations:** 1 Laboratoire de Génétique Moléculaire et d’Analyse des Génomes (LGMAG), Faculté des Sciences et Techniques, Université d’Abomey-Calavi, BP 142, Abomey-Calavi, Bénin; 2 Laboratoire de Biomathématiques et d’Estimations Forestières (LABEF), Faculté des Sciences Agronomiques, Université d’Abomey-Calavi, 04 BP 1525, Cotonou, Bénin; 3 Laboratoire d’Ecologie Appliquée (LEA), Faculté des Sciences Agronomiques, Université d’Abomey-Calavi, 01 BP 526, Tri postal, Cotonou, Bénin; 4 Regional Universities Forum for Capacity Building in Agriculture (RUFORUM), P.O. Box 16811, Wandegeya, Kampala, Uganda; 5 Herbier National, Département de Biologie végétale, Faculté des Sciences et Techniques, Université d’Abomey-Calavi, 01 BP 4521, Cotonou, Bénin

**Keywords:** Biodiversity, Quantitative ethnobotany, Prioritization, Accumulation curve

## Abstract

**Background:**

Spices have always been used for their flavor-enhancement characteristics and for their medicinal properties. In Benin, scientific research on spices is scarce, despite their importance in the local population’s daily needs. This study investigated the diversity of wild spices and documented the associated traditional knowledge that can be used for their valuation, domestication, and sustainable management in the Sudano-Guinean Zone of Benin.

**Methods:**

Data were collected during field expeditions using semi-structured interviews in ten localities across the three phytodistricts of the zone. Species richness and Shannon’s diversity index were estimated using species accumulation curves. Use report (UR), cultural importance, use value (UV) index, and informant consensus factor (*F*_ic_) were used to assess traditional knowledge on wild species, their local importance, and informants’ agreement among sociolinguistic groups. Priority wild spices were finally identified using an approach combining eight criteria (native status, economic value, ethnobotanical value, global distribution, national distribution, in-situ and ex-situ conservation status, legislation, and threats assessment) in four prioritization methods (point scoring procedure, point scoring procedure with weighting, compound ranking system, and binomial ranking system).

**Results:**

A total of 14 species, belonging to 12 genera and 9 families, were inventoried. The most prominent families were Zingiberaceae (21.43%), Annonaceae (21.43%), and Rutaceae (14.29%). More than 200 specific uses were reported, with the Tchabè people holding the greatest level of knowledge (70 uses; UR = 5.70 ± 0.33). The culturally most important spices differed among sociolinguistic groups. Most of the informants agree on the use of the species among (*F*_ic_ = 0.72–0.98) and across the considered use categories (*F*_ic_ = 0.88–0.99). The highest UV were registered for *Aframomum alboviolaceum* (UV = 0.93), *Lippia multiflora* (UV = 0.76), and *Aframomum angustifolium* (UV = 0.18). Overall, people perceived wild spices as declining due to agriculture, grazing, and drought. Five species, *A. alboviolaceum*, *L. multiflora*, *Monodora tenuifolia*, *Xylopia aethiopica*, and *Z. zanthoxyloides*, were the most prioritized for conservation.

**Conclusions:**

This study provides information relevant for the implementation of conservation and domestication actions of wild spices in Benin. Priority species could be integrated into traditional agroforestry systems (e.g., home gardens). However, for this to be effective, further research should be undertaken on morphological and genetic diversity and propagation methods of priority wild spices.

## Background

Biodiversity provides goods and services that sustain human life [1]. Yet, biodiversity is declining at unprecedented rate, which has adverse effects on human wellbeing. The quest to maintain the provisioning power of biodiversity has led to the development of multilateral environmental agreements (such as the Convention on Biological Diversity) and to increasing conservation efforts at country level aimed at slowing depletion [2]. In Benin, attention has been dedicated to the conservation of biological diversity and sustainable use of natural resources [3, 4]. So far, non-timber forest products (NTFPs) are among the most studied groups. Rural households in sub-Saharan Africa do rely on them for subsistence and trade [5]. The NTFPs are important in ensuring food security, meeting medicinal needs, and providing sources of income [6], especially in periods of drought and starvation [7]. However, owing to a changing climate [8] and anthropogenic activities such as agriculture, deforestation, overexploitation, bush fires, and livestock grazing, the NTFPs are threatened by dramatic habitat loss and changing ecologies [9].

Non-timber forest products include different taxonomic and functional groups including wild spices. A spice is “any dried, fragrant, aromatic and pungent edible vegetable or plant substance, in the whole, broken or ground form, which contributes flavor; whose primary function in food is seasoning rather than nutritional, and which may contribute relish or piquancy to foods or beverages, that is true to name and from which no portion of any volatile oil or other flavoring principle has been removed, or to which no additive or spent spice has been added” [10], except for onions, garlic, and celery [11]. Spices have long been valued for their flavor-enhancement characteristics and their medicinal properties [12]. They have proven effective in the treatment of various ailments including cough, fever, paralysis, infertility, urinary disorder, tooth ache, snakebite, menstrual disorder, and diabetes among others [13, 14]. They are also important in reducing the incidence of chronic diseases [12]. Many wild spices are rich in various bioactive compounds including alkaloids, polyphenols, flavonoids, steroids, and essential oils, which confer pharmacological potential to them. Some species are rich in key nutrients such as phosphorus, iron, calcium, and magnesium, and constitute a qualitative nutritional source for the local communities [15]. However, since several wild species are under threats of extinction, any action targeting their domestication and sustainable management is welcome.

Sustainable management of wild spices requires their documentation. This is a fundamental first step that helps in revealing the resource base for their better management. The relationship between plant species and their use by local communities is recognized as a central question in conservation science [16]. As such, understanding the determinants of the use of plants by rural communities has become a major concern in ethnobotanical studies [17, 18]. Traditional knowledge can help to clearly understand the different uses and processes developed by local communities over generations and is capital for the valuation of plant species [19]. Therefore, documenting the knowledge of local populations on wild spices is crucial as this information can help in the efficient valuation of these resources at a local level while considering different cultures across the study area.

Since financial resources for conservation activities are often limited, it is important to establish priority species to be conserved [20]. This allows conservationists to know which taxa should be primarily targeted for conservation—those that are not priority and those for which there is insufficient information to know whether or not they are priority for conservation [21]. Prioritization of wild spices is thus essential for their active conservation as it will help optimal use of available resources.

The main objective of this study was to assess the diversity of wild spices and document the associated traditional knowledge that can be used for their valuation, domestication, and sustainable management in the Sudano-Guinean zone of Benin. Specifically, the study (i) assessed the diversity of the wild spices, (ii) assessed the traditional knowledge pertaining to the species, and (iii) documented priority species deserving urgent conservation measures in the Sudano-Guinean zone of Benin.

## Material and methods

### Study area

**Fig. 1:**
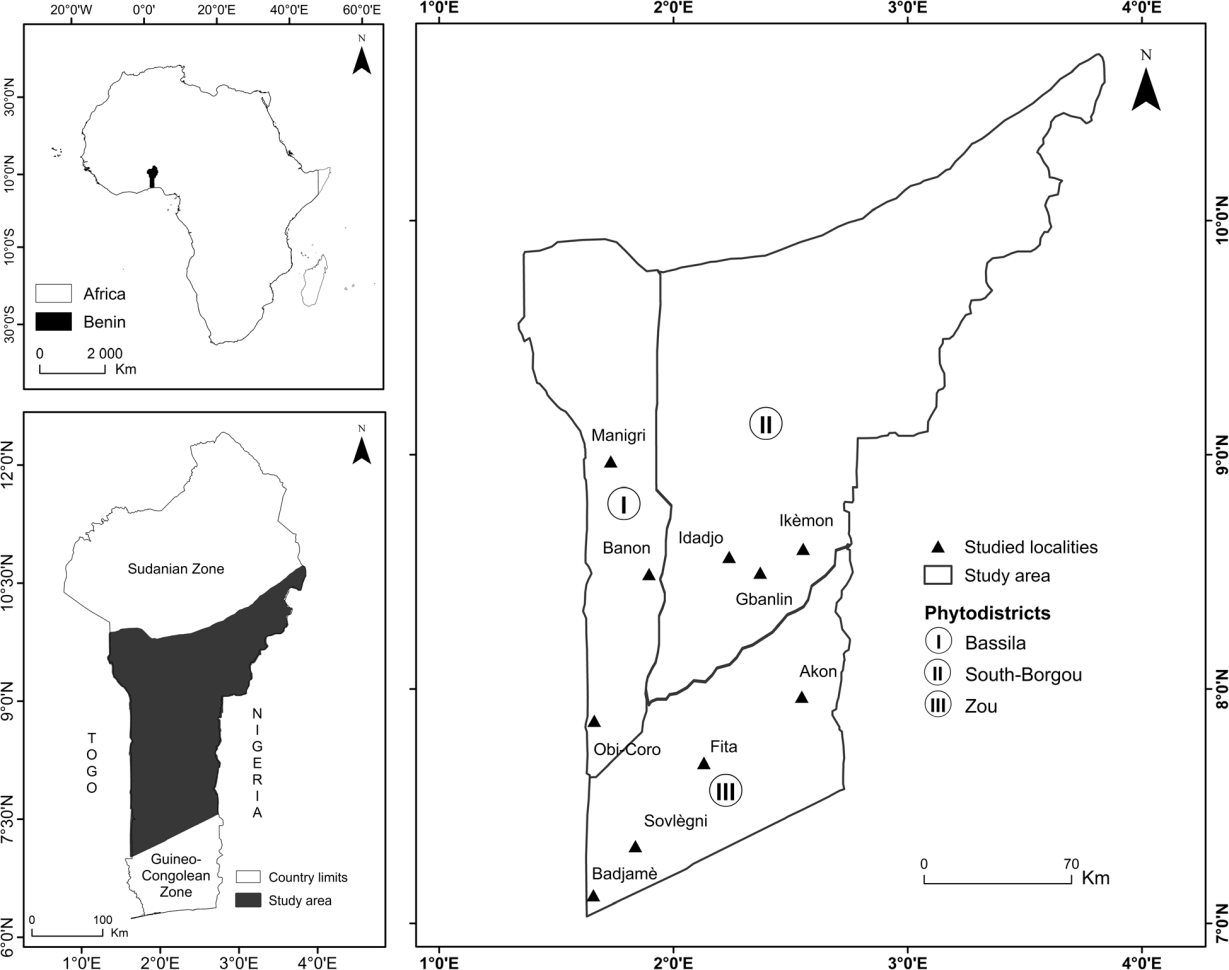
Map of the study area showing phytodistricts and surveyed villages

The study was conducted in the Sudano-Guinean zone (SGZ) of Benin (Fig. 1). The area lies between 7° 30′ and 9° 45′ N and extends from the district of Dassa to the district of Bembèrèkè. The rainfall regime is bimodal with a tendency to unimodal. The mean annual rainfall varies from 1100 to 1300 mm [22]. The relative humidity varies between 31 and 98% while the annual temperature ranges from 25 to 29 °C [23]. The zone is subdivided into three phytogeographical districts: Bassila, Zou, and South-Borgou. The phytodistrict of Bassila covers 9% of the national territory and is characterized by an annual rainfall of between 1200 and 1300 mm [24]. The soils are ferrallitic with concretions and breastplates. The vegetation is characterized by semi-deciduous forest, woodland, and riparian forest. The species richness of this phytodistrict is estimated to be 450 species with several rare plants of immense importance [25]. The phytodistrict of Zou occupies 9% of the national territory with rainfall of between 1100 and 1200 mm and ferruginous soils on crystalline rocks [24]. It is characterized by dry forest, woodland, and riparian forest which arbor a total of 350 species [25]. The phytodistrict of South-Borgou covers 22% of the country and has a unimodal rainfall averaging 1200 mm [24]. South-Borgou has ferruginous soils on crystalline rocks and its vegetation is characterized by dry forest, woodland, and riparian forest with 340 plant species [4]. The main sociolinguistic groups occupying the area are Fè, Fon, Idatcha, Mahi, Tchabè, Nago, Bariba, Anii, and Lokpa [26, 27]. The main activities of those groups are extensive agriculture, animal husbandry, and uncontrolled exploitation of woodlands and gallery forests [28].

### Sampling

The presence of wild spices across Benin was reported by Akoègninou et al. [29]. A brief preliminary survey was undertaken in November 2015 throughout ten localities randomly selected in the study area. Uses by diversity of sociolinguistic group and the effective presence of wild spices was used as the main criteria in selecting localities in each phytodistrict. Random selection was used after selecting localities that met these criteria. In each locality, 30 informants [30] were asked if they knew or made use of at least one wild spice in their household. The proportion *p* of positive answers was used to compute the sample size *n* for each locality using the normal approximation of the binomial distribution [30]:

1 $$ n=\frac{U_{1-\alpha /2}^2\times p\left(1-p\right)}{d^2}, $$

with:

- *U*_1 *− α/*2_ the value of the normal random variable corresponding to a probability value of 1 *− α/*2. For a probability value of 0.975 (or *α* = 0.05), *U*_1 *− α/*2_ = 1.96
- *d* the margin error of the estimation of any parameter to be computed from the survey and was fixed at 0.08

From the formula above, the number of informants randomly considered throughout the study area (all localities considered) was 218 informants including 108 women (Table 1). Ethnobotanical surveys were carried out during 6 months from March to August 2016.

**Table 1:** Sample size by locality, sociolinguistic group, phytodistrict and gender

Country district	Locality	SG	Sample size (locality)			PD	Sample size (PD)		
			W	M	Total		W	M	Total
Aplahoué	Badjamè	Adja	9	11	20	Zou	51	46	97
Djidja	Sovlègni	Fon	18	19	37				
Dassa	Fita	Idatcha	13	7	20				
Savè	Akon	Tchabè	11	9	20				
Ouessè	Ikèmon	Nago	9	11	20	South Borgou	31	29	60
Ouessè	Gbanlin	Mahi	10	10	20				
Ouessè	Idadjo	Nago	12	8	20				
Bantè	Banon	Itcha	6	15	21	Bassila	26	35	61
Savalou	Obi-Coro	Ifè	8	12	20				
Bassila	Manigri	Tchabè	12	8	20				
Total			108	110	218		108	110	218

*SG* sociolinguistic group, *W* women, *M* men, *PD* phytodistrict

### Data collection

Data were collected through field exploration and individual semi-structured interviews using questionnaire. The questionnaire was twofold: the first part assessed sociodemographic characteristics of informants (name, sex, age, sociolinguistic group, and main activity) while the second part was concerned with their knowledge on wild spices. Each informant was asked to list, using his local name, the wild spices that he (or she) knew and/or used. For each species, data on the meaning of the name, the category of use (food, medicinal, ceremony, cultural, other), the plant parts used, the description of uses, the main habitats where the species is encountered, the local perception of its population dynamic over time (declining, stable, or increasing), the perception of the nutritional value of the species, and its economic value (low, average and high) were gathered during the surveys. Interviews were recorded and conducted entirely in the local language of the informants, with the help of a translator when and where necessary. Individual semi-structured questionnaire interviews were followed by direct observations on the field (home garden, field, fallow, savanna, or forest) accompanied by key informants to observe the species in their natural habitat or in cultivation while minimizing the risks of erroneous identifications. The species were photographed, collected, and pressed for identification in the herbarium [31]. Literature was used to collect data regarding the species origin, its global and national distribution, its conservation status (in situ and ex situ), existence of legislation, and threat assessment, for prioritization purpose. Scientific articles, the flora of Benin [29], the Biodiversity Atlas for West Africa [25], the IUCN online database [32], the Red list of threatened plant species in Benin [4], and the online database of the Plant Resources of Tropical Africa [33] were used as complementary sources of data.

### Data analysis

Collected specimens were identified at species taxonomic level and their life form checked using the illustrated reference book of Arbonnier [34], the Analytic Flora of Benin [29], and the expertise of a specialist of the National Herbarium of Benin. The chorological type of each species was determined following White [35]. Relative frequencies were computed by species, botanical family, life form, and chorological type in order to generate tables and bar plots at levels of phytodistricts and sociolinguistic groups. To estimate wild spices richness, species compositions as listed by respondents were translated into a presence-absence matrix by phytodistrict and species accumulation curve was generated based on the first-order Jackknife method and 100 permutations, using *EstimateS* version 9.1.0 software for Macintosh [36]. The first-order Jackknife is a non-parametric incidence-based estimator that gives an accurate approximation of species richness [37]. Diversity of wild spices in each phytodistrict was assessed using Shannon’s diversity index computed in *EstimateS* also using the species accumulation curve. To compare the estimated wild spices richness and diversity among phytodistricts, Kruskal-Wallis test was applied since assumptions of normality and homogeneity of variance were violated [38]. In addition, the Dunn post hoc test was performed. The Dunn post hoc test is appropriate for groups with unequal sizes [39]. Differences in wild spices composition among phytodistricts as cited by respondents were tested using analysis of similarities (ANOSIM) [40]. ANOSIM was based on Jaccard’s dissimilarity distance using 1000 permutations. To describe probable relationship between the species and their habitats as locally mentioned, a correspondence analysis (CA) was performed on the contingency table with species in rows and habitats in columns.

Traditional knowledge was assessed through calculation of the use report (UR) [41] which is the number of uses reported by a given informant. Since the UR values were not over-dispersed, a generalized linear model (GLM) with Poisson error distribution was applied to assess their variation according to phytodistrict, sociolinguistic group, gender, age category, main activity, and education level. The model that best fits with the data was selected by combining Akaike information criteria (AIC) and Bayesian information criteria (BIC). To assess the homogeneity in the information provided by the respondents, the informant consensus factor (*F*_ic_) was calculated for each use category and across use categories, using the following formula [42]:

2 $$ {F}_{\mathrm{ic}}=\frac{n_{\mathrm{ur}}-{n}_{\mathrm{t}}}{n_{\mathrm{ur}}-1}, $$

Where:

- *F*_ic_ = informant consensus factor
- *n*_ur_ = number of use reports from informants for a particular plant use category
- *n*_t_ = number of taxa that are used for that plant use category for all informants.

*F*_ic_ values range between 0 and 1. Values close to 0 indicate that the plants are chosen randomly in the use category, or the informants do not exchange information about their use. High *F*_ic_ values (near 1) indicate that the plants are chosen based on a well-defined criterion in the community and/or the information is shared among informants [43].

Cultural importance of the wild spices was assessed using the cultural importance value from both individual informant and community perspectives as in Paniagua Zambrana et al. [44]. The cultural importance value from individual informant perspective was calculated as follow [41]:

3 $$ \mathrm{CI}={\sum}_{u={u}_1}^{u_{\mathrm{NC}}}{\sum}_{i={i}_1}^{i_N}{\mathrm{UR}}_{ui}/N, $$

where:

- UR_ui_ = total number of informants reporting a species within a particular use category
- NC = total number of use categories
- *N* = total number of informants.

Community cultural importance was calculated for each species following Paniagua Zambrana et al. [44] as the sum across all use categories of the number of communities reporting the species divided by the total number of communities surveyed.

To assess the importance of each wild spice, the use value (UV) index modified by Rossato et al. [45] from Phillips and Gentry [46] as follow:

4 $$ UV=\sum U/n, $$

where:

- *UV* = use value of a species
- *U* = number of quotations per species
- *n* = number of informants.

For defining priority wild spices for conservation, the approach proposed by Magos Brehm et al. [21] and recently applied by Idohou et al. [47] to the Crop wild relatives in Benin was used. Eight criteria were used: (i) native status, (ii) economic value, (iii) ethnobotanical value, (iv) global distribution, (v) national distribution, (vi) in-situ and ex-situ conservation status, (vii) legislation, and (viii) threats assessment (IUCN redlist). The method consisted in combining four different prioritization setting methods that used the aforementioned criteria: Point Scoring Procedure (PSP), Point Scoring Procedure with Weighting (PSPW), Compound Ranking System (CRS), and Binomial Ranking System (BRS). In PSP, the inventoried species were given a series of scores for each criterion (Table 2). Then, the overall score was obtained for each wild spice by adding the scores of individual criteria. Species with a greater value have higher conservation needs. PSWP is comparable to PSP, except that here a specific weight was assigned to each criterion (Table 2). The CRS method is based on the individual criterion ranking positions, which were combined to obtain a compound rank for each species (Table 3). BRS uses a series of questions with binomial answers (i.e., “yes” or “no”). The *yes* answer (1) was always given more priority than the *no* (0). A sub-list of eight priority wild spices was established for each method. The number of times that the same species appeared on each sub-list was marked, and each species was given an overall score. The five species that have received the greater overall scores were given higher priority. In the case where many species had the same overall score, cultural importance and socio-economic considerations were used to separate the species.

**Table 2:** Score attribution in the point scoring procedure (PSP) and point scoring procedure with weighting (PSPW) (adapted from Magos Brehm et al. [21])

Criteria	Evaluation criteria	Score attribution (PSP)	PSPW weight (%)
Species origin	(a) Native, (b) exotic, (c) doubtfully native, (d) no data	(a) 4; (b) 3; (c) 2; (d) 1	15
Economic value	(a) high, (b) average, (c) Low, (d) no data	(a) 4; (b) 3; (c) 2; (d) 1	10
Ethnobotanical value	(a) 19–21; (b) 16–18; (c) 13–15; (d) 10–12; (e) 7–9; (f) 4–6; (g) 1–3; (h) No known uses	(a) 7; (b) 6; (c) 5; (d) 4; (e) 3; (f) 2; (g) 1; (h) 0	20
Global distribution	(a) WA, (b) WA + 1 region, (c) WA + 2 regions, (d) WA + 3 regions, (e) Africa, (f) worldwide, (g) no data	(a) 6; (b) 5; (c) 4; (d) 3; (e) 2; (f) 1; (g) 0	15
National distribution	(a) 1; (b) 2; (c) 3; (d) 4; (e) 5; (f) 6; (g) 7; (h) 8; (i) 9; (j) 10; (k) no data	(a) 10; (b) 9; (c) 8; (d) 7; (e) 6; (f) 5; (g) 4; (h) 3; (i) 2; (j) 1; (k) 0	7.5
Conservation status	(a) In situ, (b) ex-situ, (c) other, (d) no data	(a) 4; (b) 3; (c) 2; (d) 1	10
Legislation	(a) international, (b) national, (c) locale, (d) no data	(a) 4; (b) 3; (c) 2; (d) 1	7.5
Threatened status	(a) CR, (b) EN, (c) VU, (d) NT, (e) LC, (f) DD, (g) NE	(a) 7; (b) 6; (c) 5; (d) 4; (e) 3; (f) 2; (g) 1	15

*WA* West Africa, *CR* critically endangered, *EN* endangered, *VU* vulnerable, *NT* near threatened, *LC* least concern, *DD* data deficient, *NE* not evaluated

**Table 3:** Rank attribution in the compound ranking system (CRS) method (adapted from Magos Brehm et al. [21])

Criteria	Rank of sub-criteria										
	R1	R2	R3	R4	R5	R6	R7	R8	R9	R10	R11
Species origin	Native	Exotic	Doubtfully native	No data	–	–	–	–	–	–	–
Economic value	High	Average	Low	No data	–	–	–	–	–	–	–
Ethnobotanical value	20	19	18	17	16	15	14	13	12	No data	–
Global distribution	WA	WA + 1 region	WA + 2 regions	WA + 3 regions	Africa	World	No data	–	–	–	–
National distribution	1	2	2	4	5	6	7	8	9	10	No data
Conservation status	in situ	ex situ	Other	No data	–	–	–	–	–	–	–
Legislation	International	National	Local	No data	–	–	–	–	–	–	–
Threatened status	CR	EN	VU	NT	LC	DD	NE	–	–	–	–

*WA* West Africa, *CR* critically endangered, *EN* endangered, *VU* vulnerable, *NT* near threatened, *LC* least concern, *DD* data deficient, *NE* not evaluated

All analyses were conducted in the statistical software R 3.4.1 [48]. The Dunn hoc test was performed using the package *FSA* [49], the ANOSIM analysis in the *vegan* package [50], and the correspondence analysis in the package *FactomineR* [51].

## Results

### Diversity and richness of wild spices

Estimation of wild spices richness indicated 14 species in the Sudanian zone (CI 13.83–14.32) belonging to 9 families and 12 genera (Table 4). The three most important families were Zingiberaceae (3 species), Annonaceae (3 species), and Rutaceae (2 species). The remaining 6 families (Lamiaceae, Poaceae, Verbenaceae, Piperaceae, Polygonaceae, and Balanophoraceae) were represented by only one species each. The species were composed of herbs (42.86%), shrubs (28.37%), trees (21.43%), and liana (7.14%).

**Table 4:** General characteristics of the wild spices inventoried

N°	Species	Family	Vernacular name (sociolinguistic group)	LF	PT	Habitat	PU	PA	Use categories	LF
1	*Aeollanthus pubescens* Benth.	Lamiaceae	Fé okuta, Kpon’kouta (Tchabè)	H	S	7	lf, fw, wp	RS	foo, med	H
2	*Aframomum alboviolaceum* (Ridley) K.Schum.	Zingiberaceae	Gbétakin (Fon); Koutchou (Adja); Atalè okou, Ibouro (Idatcha); Ebouro, Ibio, Bobota (Ifè, Tchabè); Okpogloé (Mahi); Ebo (Itcha)	H	AT	3, 4, 5, 6	lf, rt., st, fr, sd	DS	foo, med, fod, oth	H
3	*Aframomum angustifolium* (Sonn.) K.Schum.	Zingiberaceae	Tchankoko (Ifè, Tchabè)	H	GC	3, 4, 6	lf, rt., st, fr	RS	foo, med, cult, oth	H
4	*Aframomum melegueta* (Roscoe) K.Schum.	Zingiberaceae	Takù (Adja)	H	GC	5, 6	lf, rt., sd	NA	med, cult	H
5	*Clausena anisata* (WilId.) Hook.f. ex Benth.	Rutaceae	Azizinma, Gbozohouin, Tchakatouma (Fon); Ewé kikani (Idatcha)	Sh	SG	3, 7	lf, rt	RS	med, cult	S
6	*Cymbopogon giganteus* (Hochst.) Chiov.	Poaceae	Oflin (Idatcha); gbézin (Fon)	H	AT	3, 7	lf	AS	foo, med, fod	H
7	*Lippia multiflora* Moldenke	Verbenaceae	Nyenya, aglàla (Fon, Mahi); Aglàla, Aklala sê (Mahi); Kanhoun (Idatcha); Tchagara (Ifè); Tchagà (Itcha); Kinhoun kinhoun, Kanhoun kanhoun (Tchabè)	Sh	S	1, 2, 3, 4, 6, 7	lf, fw, st, rt., wp	AS	foo, med, cult	S
8	*Monodora tenuifolia* Benth.	Annonaceae	Ariwo (Idatcha)	T	GC	7	rt, bk, sd	RS	foo, med, cult	T
9	*Piper guineense* Schumach. & Thonn.	Piperaceae	Kanlin man, lènlènkoun (Adja)	L	AT	4, 5	lf, fr, st, rt	AS	foo, med, cult	L
10	*Securidaca longipedunculata* Fresen.	Polygonaceae	Attakpa wanon, Attakpa wanhuin huin, Kpatalè (Fon, Idatcha)	Sh	AT	3, 4, 7	rt, bk	AS	med	S
11	*Thonningia sanguinea* Vahl	Balanophoraceae	Atin madodè (Idatcha); Otchoulélé (Ifè)	H	AT	3, 4, 6	rt	RS	med	H
12	*Uvaria chamae* P.Beauv.	Annonaceae	Yaha (Idatcha); Yalaha (Fon)	Sh	SGC	3, 4, 7	lf, fr, rt	AS	foo, med	S
13	*Xylopia aethiopica* (Dunal) A.Rich.	Annonaceae	Esso (Adja)	T	AT	5	fr	AS	med, cult	T
14	Zanthoxylum zanthoxyloides (Lam.) Zepernick & Timler	Rutaceae	Tchanouwèlè (Itcha); Sanouyèlè (Tchabè)	T	SG	1, 2, 3, 4	rt	AS	foo, med	T

*LF* life form, *PT* phytogeographical type, *PA* period of availability, *H* herb, *L* liana, *Sh* shrub, *T* tree, *AT* afrotropical, *GC* Guineo-Congolian, *S* Sudanian, *SG* Sudano-Guinean, *SGC* Sudano-Guineo-Congolian, *lf* leaf, *fw* flower, *wp* whole plant, *rt*. root, *st* stem, *sd* seed, *fr* fruit, *bk* bark, *cer* ceremony, *med* medicine, *foo* food, *cult* cultural, *oth* other, *DS* dry season, *AS* all season, *RS* rainy season, *1* home garden, *2* field, *3* savanna, *4* natural forest, *5* sacred forest, *6* wetland, *7* hill

**Fig. 2:**
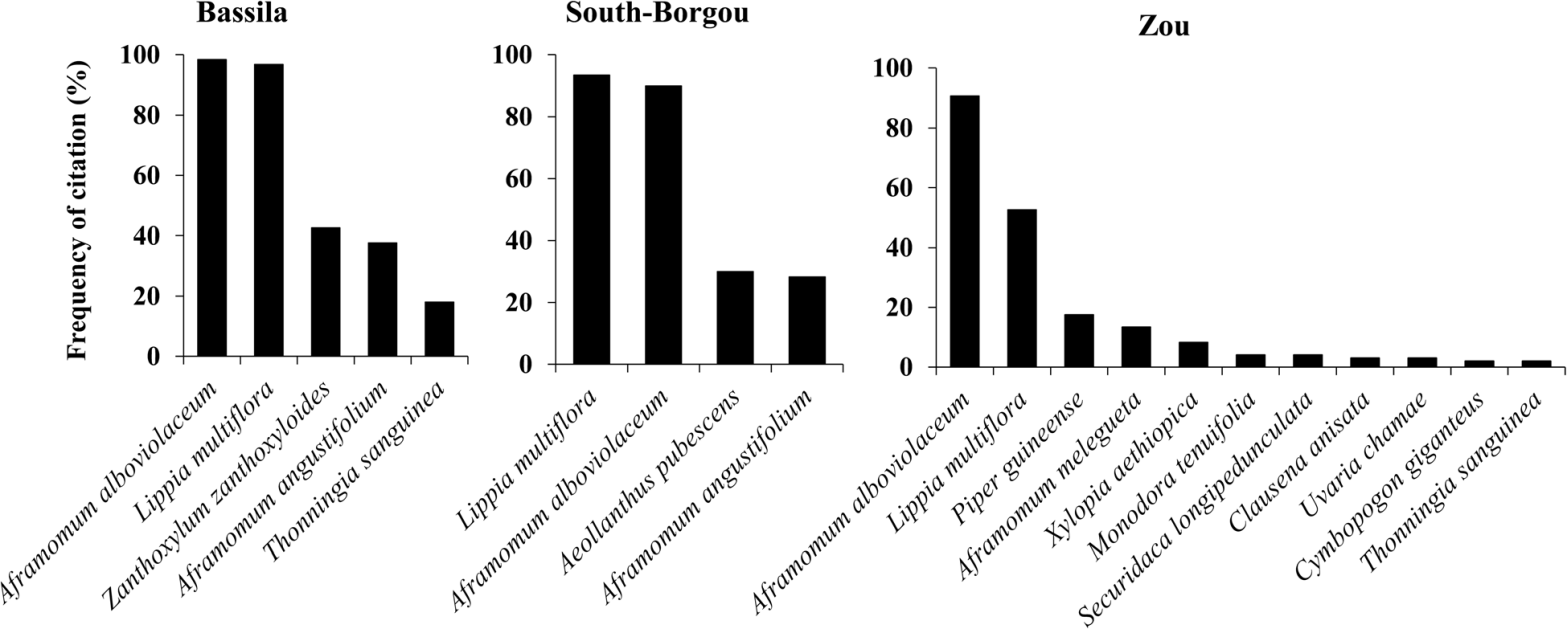
Wild spices encountered in the Sudano-Guinean zone of Benin. **a** Bassila. **b** South-Borgou. **c** Zou phytodistricts

**Fig. 3:**
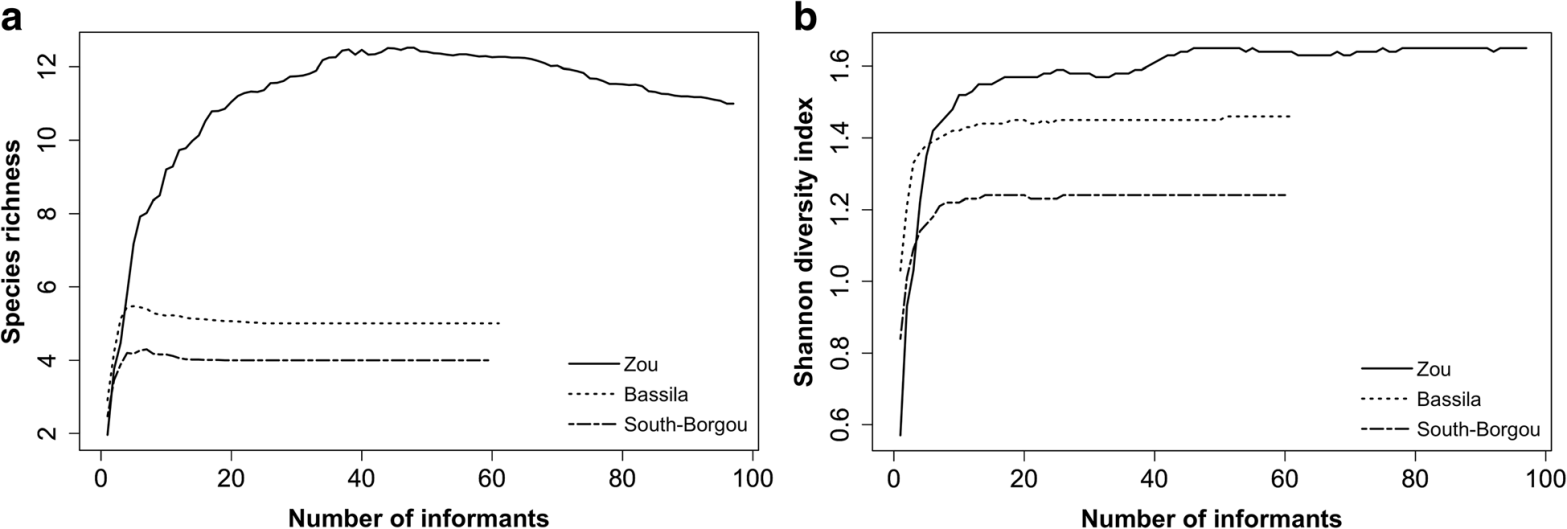
Estimated species richness (**a**) and Shannon’s diversity (**b**) for the species based on incidence data

Estimation of species richness by phytodistrict revealed 11 species (CI 10.78–11.54) belonging to 8 families in Zou, 5 species (CI 4.94–5.01) belonging to 4 families in Bassila, and 4 species (CI 3.93–4.05) belonging to 4 families in South-Borgou (Fig. 2). The first-order Jackknife estimator overestimated wild spices richness as the sampling started and then decreased slightly with increasing sample size (Fig. 3a). Comparison of estimated richness among phytodistricts indicated that wild spices richness was significantly higher in Zou than in Bassila and South-Borgou respectively (Fig. 3a; *p* < 0.001). Similarly, the Shannon diversity index was significantly higher in Zou (1.58) than Bassila (1.43) and South-Borgou (1.22) respectively (Fig. 3b; *p* < 0.001). Therefore, the phytodistrict of Zou was the richest and most diversified in terms of used wild spices.

There were significant differences in the composition of wild spices used by respondents among phytodistricts (ANOSIM; *R* = 0.123, *p* = 0.001). Coefficient of similarity between Zou and South-Borgou (0.15, *p* = 0.003) and the one between Zou and Bassila (0.23, *p* ≤ 0.001) were low. This reveals that the people in the phytodistricts of Zou knew and used wild spices differently from those of the people from South-Borgou and Bassila respectively. Conversely, the coefficient of similarity between Bassila and South-Borgou was 0.50 (*p* ≤ 0.001), indicating that half of the species mentioned as wild spices by people from Bassila were also known by informants from South-Borgou, and vice-versa.

**Fig. 4:**
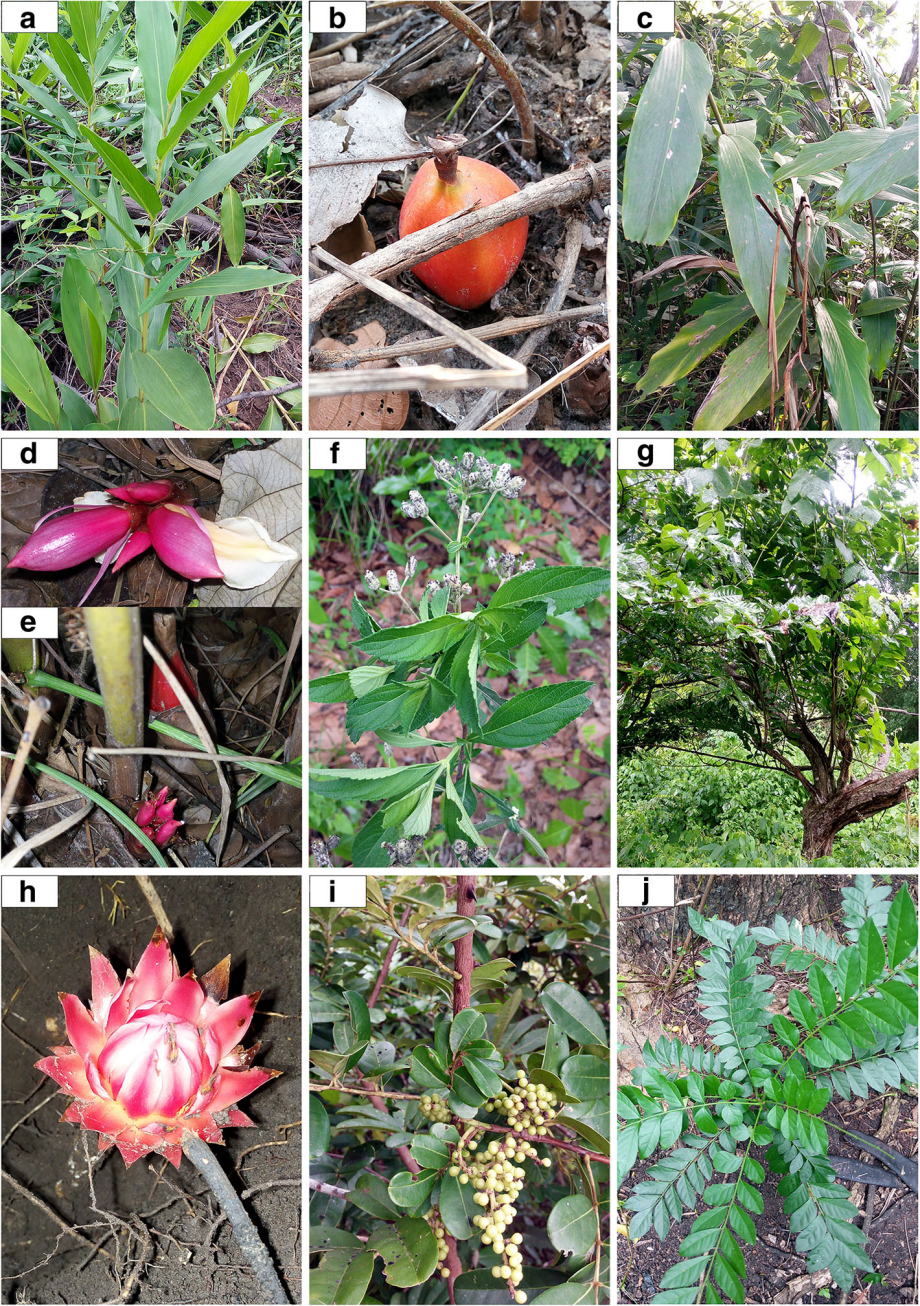
Pictures of selected wild spices in the Sudano-Guinean zone of Benin. **a** Leaves and **b** fruit of *Aframomum alboviolaceum*. **c** Leaves, **d** flower, and **e** fruit of *Aframomum angustifolium.* **f** Leaves and flowers of *Lippia multiflora*. **g** Tree *Monodora tenuifolia*. **h** Flower of *Thonningia sanguinea.* **i** Leaves and unripe fruits of *Zanthoxylum zanthoxyloides*. **j** Leaves of *Clausena anisata*

**Fig. 5:**
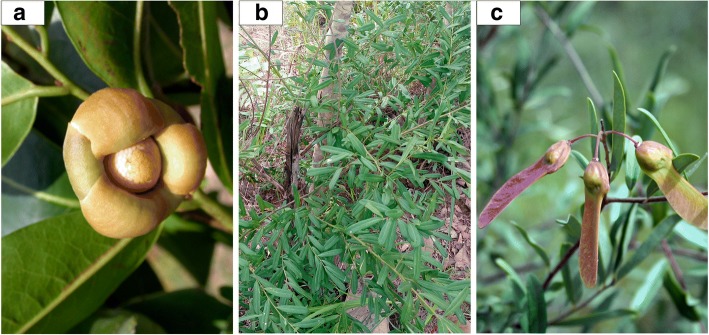
Pictures of selected wild spices in the Sudano-Guinean zone of Benin. **a** Flower of *Uvaria chamae.* **b** Leaves and **c** fruits of *Securidaca longipedunculata*

The distribution of the spices according to their chorological type showed the predominance of Afrotropical (42.86%) and Guineo-Congolian (21.43%) species whereas Sudanian (14.29%), Sudano-Guinean (14.29%), and Sudano-Guinean (7.13%) elements were poorly represented (Table 4). Vernacular names were diverse and varied according to the species but also among areas as well as within the same sociolinguistic area (Table 4). Indeed, 42 vernacular names were recorded for the 14 investigated wild spices. The analysis of the meanings of vernacular names in each dialect (Table 5) revealed that the wild spices were named essentially based on their morphological traits, habitats, taste, habits, smell, or origin. However, for some species, the meanings of the vernacular names were unknown to local people. Occasionally, different sociolinguistic groups, often occupying the same geographical area share the same vernacular names for a given species. Pictures of selected wild spices are presented in Figs. 4 and 5.

**Table 5:** List of vernacular names recorded per species and their meaning

Species	Vernacular names	Sociolinguistic groups	Meaning of the vernacular names
*Aeollanthus pubescens*	Fé okuta, Kpon’kouta	Tchabè	Grow on the hills
*Aframomum alboviolaceum*	Gbétakin	Fon	Bush pepper
	Gbétakoun	Fon	*Aframomum melegueta* of the bush
	Koutchou	Adja	–
	Atalè okou	Idatcha	Bush pepper
		Ibouro	–
	Ebouro	Ifè	Red fruit
	Ibio	Nago	Red fruit
	Bobota	Tchabè	Red fruit emerging from the ground
			Pungent seeds like *A. melegueta*’s ones
	Okpogloé	Mahi	Fruits emerging from the ground
	Ebo	Itcha	Red fruit
*Aframomum angustifolium*	Tchankoko	Ifè, Nago Tchabè,	–
	Sankoko		–
*Aframomum melegueta*	Takù	Adja	–
*Clausena anisata*	Azizinma	Fon	Repel the ants
	Tchakatouma	Fon	Leaves used for making magic to counter tchakatou dark magic
	Gbozohouin	Fon	–
	Ewé kikani	Idatcha	–
*Cymbopogon giganteus*	Oflin	Idatcha	–
	Gbézin	Fon	–
*Lippia multiflora*	Nyenya	Fon	–
	Aglàla	Fon, Mahi	–
	Aklala sê	Mahi	Plant with aromatic flower introduced from Accra (Ghana)
	Kanhoun	Idatcha	–
	Tchagara	Ifè	Mint flavor
	Tchagà	Itcha	–
	Kinhoun kinhoun	Nago	Pleasant flavor
	Kanhoun kanhoun	Tchabè	Pleasant flavor
*Monodora tenuifolia*	Ariwo	Idatcha	–
*Piper guineense*	Kanlin man	Adja	Liana habit
	lènlènkoun	Adja	–
*Securidaca longipedunculata*	Attakpa wanon, Attakpa wanhuin huin	Fon	Persistent scent of the root’s bark
	Kpatalè	Idatcha	–
*Thonningia sanguinea*	Atin madodè	Idatcha	Leafless plant
	Otchoulélé	Ifè	The flowers appear with the new moon
*Uvaria chamae*	Yaha	Idatcha	–
	Yalaha	Fon	
*Xylopia aethiopica*	Esso	Adja	–
*Zanthoxylum zanthoxyloides*	Tchanouwèlè	Itcha	Pungent taste and pleasant flavor
	Sanouyèlè	Tchabè	Pungent taste and pleasant flavor

### Local perception on the distributional pattern of species across habitat types

**Fig. 6:**
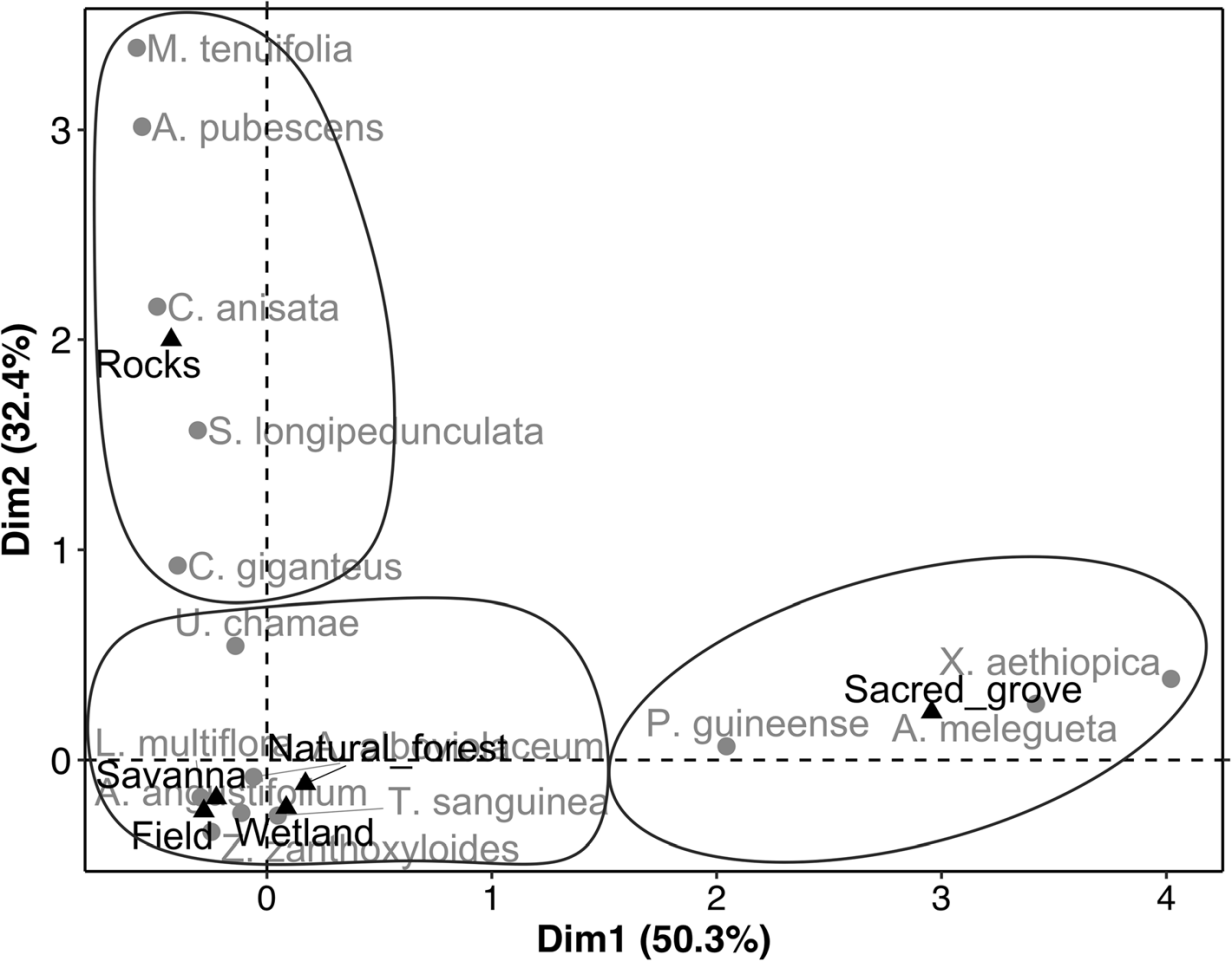
Projection of the species and types of habitat in the correspondence analysis axes systems

Result of the CA performed on the wild spices and their habitats (with 82.62% of the observed variation saved on the first two axes) indicated that *Xylopia aethiopica* (Dunal) A.Rich., *Piper guineense* Schumach. & Thonn., and *Aframomum melegueta* (Roscoe) K.Schum. were mainly reported as occurring in sacred groves while *Zanthoxylum zanthoxyloides* (Lam.) Zepernick & Timler, *Lippia multiflora* Moldenke, *Aframomum angustifolium* (Sonn.) K.Schum., *Aframomum alboviolaceum* (Ridley) K.Schum., *Thonningia sanguinea* Vahl, and *Uvaria chamae* P.Beauv. were reported to mainly occur in savanna, home gardens, fields, natural forests, or wetlands (axis 1; Fig. 6). *Aeollanthus pubescens* Benth., *Monodora tenuifolia* Benth., *Clausena anisata* (Willd.) Hook.f. ex Benth., *Securidaca longipedunculata* Fresen., and *Cymbopogon giganteus* (Hochst.) Chiov. were reported as occurring on rocky soils and hills (axis 2; Fig. 6).

### Traditional knowledge and cultural importance of the wild spices

#### Traditional knowledge (TK) on the wild spices

Traditional knowledge (TK) on wild spices varied (*p* < 0.05) according to phytodistricts, sociolinguistic groups and gender. No variation (*p* > 0.05) of TK was observed with respect to age category, main activity, or education level. Informants reported the same number of uses of wild spices in both Bassila (UR = 4.59 ± 0.22) and South-Borgou (UR = 4.05 ± 0.17) phytodistricts while less uses were reported in Zou phytodistrict (UR = 3.13 ± 0.18). As regards variation according to the gender, men reported more uses than women (UR = 4.09 ± 0.18 and UR = 3.46 ± 0.14 respectively). For sociolinguistic group variation, Tchabè in Bassila held the highest level of traditional knowledge (UR = 5.70 ± 0.33) followed by Itcha (UR = 4.38 ± 0.40) and Ifè (UR = 3.70 ± 0.24) sociolinguistic groups respectively. In South-Borgou phytodistrict, the Nago reported more uses (UR = 4.13 ± 0.23) than the Mahi people (UR = 3.75 ± 0.19). In Zou phytodistrict, the Idatcha reported more uses (UR = 4.62 ± 0.59) than the other groups. The number of use reports was similarly low for the Adja, Fon, and Tchabè sociolinguistic groups (UR values of 3.04 ± 0.40, 2.69 ± 0.29, and 3.06 ± 0.21 respectively).

#### Informants consensus factors (*F*_ic_) among and across use categories

Overall, a great consistency was observed among the informants regarding the uses of the wild spices, with *F*_ic_ values varying from 0.72 to 0.98 (Table 6). Food use category had the highest *F*_ic_ value 0.98 with 434 use reports for 10 plant species. The species responsible for this high value were *Aframomum alboviolaceum* and *Lippia multiflora* with 201 (24.78%) and 151 (18.62%) reported uses respectively. This category of use was followed by medicine (*F*_ic_ = 0.96; 304 use reports, 14 species), culture (*F*_ic_ = 0.95; 21 use reports, 2 species), and other uses (*F*_ic_ = 0.84; 26 use reports, 5 species). Other uses included food wrapping, fodder, rope for binding luggage in the field, and whip for punishing children. Similarly, the species responsible of the high number of reported uses within medicinal category were *L. multiflora* with 137 out of the 304 medicinal use reports (45.07%) and *A. alboviolaceum* with 57 use reports (18.75%). The least agreement between informants (*F*_ic_ = 0.72; 26 use reports, 8 species) was found for wild spices used for ceremony (Table 6). As regards the informants’ consensus across the use categories, high degree of agreement (*F*_ic_ ranged from 0.97 to 0.99) was observed across the phytodistricts (Table 7). A similar trend was found between gender with *F*_ic_ = 0.97 for both women and men. As for the sociolinguistic groups, the highest agreement (*F*_ic_ = 0.98) among information given by the informants was found for Nago and Tchabè people from Zou phytodistrict (Table 7). The least agreement (*F*_ic_ = 0.88) was found for Idatcha sociolinguistic group.

**Table 6:** Informant consensus factor (*F*_ic_) by use category

Use categories	*n*_ur_	% ur	*n*_t_	% taxa	*F*_ic_^#^
Food	434	53.45	10	71.43	0.98
Medicine	304	37.44	14	100.00	0.96
Cultural	21	2.59	2	14.29	0.95
Ceremony	26	3.20	8	57.14	0.72
Other uses	26	3.20	5	35.71	0.84
Total	811	99.88	14*	100.00*	

*n*_*ur*_ number of use report, *ur* use report, *n*_*t*_ number of taxa, *F*_*ic*_ informant consensus factor

*A taxon may be listed in many of the categories of use

^#^A high *Fic* value indicates a high level of agreement among the informants regarding wild spices used for the corresponding use category; a low value indicates a low degree of agreement

**Table 7:** Informant consensus factor (Fic) across use categories

	*n*_ur_*	% ur	*N*	*n*_t_	% taxa	*F*_ic_^#^
Phytodistrict						
Bassila	280	34.57	61	5	35.71	0.99
South-Borgou	239	29.51	60	4	28.57	0.99
Zou	291	35.93	97	11	78.57	0.97
Sex						
Men	478	59.01	110	14	100	0.97
Women	332	40.99	108	12	85.71	0.97
Sociolinguistic group						
Adja	70	8.64	20	4	28.57	0.96
Fon	94	11.6	37	8	57.14	0.92
Idatcha	60	7.41	20	8	57.14	0.88
Ifè	74	9.14	20	4	28.57	0.96
Itcha	92	11.36	21	4	28.57	0.97
Mahi	90	11.11	20	4	28.57	0.97
Nago	161	19.88	40	4	28.57	0.98
Tchabè 1	55	6.79	20	2	14.29	0.98
Tchabè 2	114	14.07	20	4	28.57	0.97

*n*_*ur*_ number of use report, *ur* use report, *N* number of informants, *n*_*t*_ number of taxa, *F*_*ic*_ informant consensus factor

*A taxon may be listed in several categories of use

^#^A high *Fic* value indicates a high level of agreement among the informants regarding wild spices used for the corresponding use category; a low value indicates a low degree of agreement

#### Cultural importance (CI) of the wild spices

**Fig. 7:**
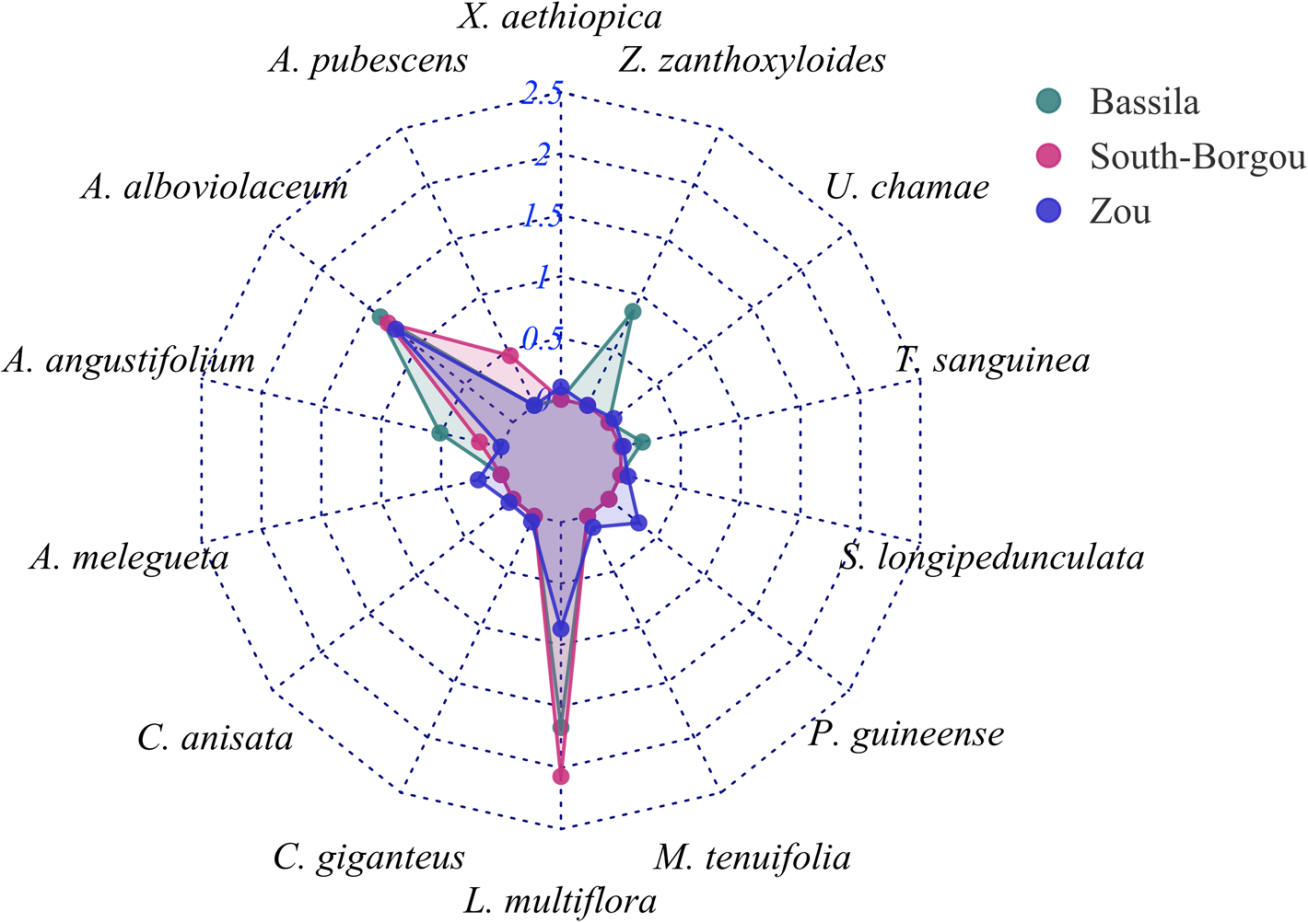
Radar plot of total CI value for each wild spice according to the phytodistrict

Based on the cultural importance index at informant level, the most culturally important wild spices in Bassila phytodistrict were *L. multiflora* (CI = 1.67), *A. alboviolaceum* (CI = 1.38), *Z. zanthoxyloides* (CI = 0.85), and *A. angustifolium* (CI = 0.51). In South-Borgou, *L. multiflora* (CI = 2.07) and *A. alboviolaceum* (CI = 1.30) were identified whereas *A. alboviolaceum* (CI = 1.22) and *L. multiflora* (CI = 0.87) were identified for Zou phytodistrict (Fig. 7). Therefore, people valued almost the same wild spices in all the Sudano-Guinean zone except in Bassila where two species (*Z. zanthoxyloides* and *A. angustifolium*) were valued in addition. The most relevant categories of use were food followed by medicine for the three phytodistricts (Table 8).

**Table 8:** Cultural importance index (CI, informant based) of wild spice use categories by phytodistrict

Use category*	Phytodistrict		
	Bassila	South-Borgou	Zou
Food	0.52	0.52	0.14
Medicine	0.37	0.35	0.10
Cultural	0.00	0.09	0.00
Ceremony	0.01	0.00	0.02
Other use	0.02	0.04	0.01
Total CI	0.92	1.00	0.27

*Average number of wild spices inventoried in each phytodistrict: 5 in Bassila, 4 in South-Borgou, and 11 in Zou

*L. multiflora* was the common most culturally important wild spice for Mahi (CI = 3.20), Tchabè from both Bassila (CI = 1.90) and Zou (CI = 1.50) phytodistricts, Nago (CI = 1.66), Itcha (CI = 1.57), Ifè (CI = 1.55), and Idatcha (CI = 0.95) sociolinguistic groups, while it had relatively less importance for Fon (CI = 0.78) and absolutely no importance for Adja. Similarly, *A. alboviolaceum* was highly valued by all sociolinguistic groups. *Z. zanthoxyloides* was most importantly valued by Tchabè from Bassila phytodistrict (CI = 1.90) and lesser by Itcha (CI = 0.66). *P. guineense* and *A. melegueta* were valued only by Adja (CI = 1.10 and 0.80 respectively), whereas *M. tenuifolia* had cultural importance only for Idatcha (CI = 0.50), and *A. angustifolium* for Itcha (CI = 0.81) and Tchabè informants from Bassila phytodistrict (CI = 0.60; Table 9).

**Table 9:** Cultural importance index (informant based) of each species according to the different sociolinguistic groups

Species	Sociolinguistic group								
	Adja	Fon	Idatcha	Ifè	Itcha	Mahi	Nago	Tchabè1	Tchabè2
*Aeollanthus pubescens*	0.00	0.00	0.00	0.00	0.00	0.00	0.68	0.00	0.00
*Aframomum alboviolaceum*	1.10	1.35	0.85	1.50	1.33	1.20	1.47	1.25	1.30
*Aframomum angustifolium*	0.00	0.00	0.00	0.10	0.81	0.00	0.29	0.00	0.60
*Aframomum melegueta*	0.80	0.06	0.00	0.00	0.00	0.00	0.00	0.00	0.00
*Clausena anisata*	0.00	0.06	0.10	0.00	0.00	0.00	0.00	0.00	0.00
*Cymbopogon giganteus*	0.00	0.00	0.25	0.00	0.00	0.00	0.00	0.00	0.00
*Lippia multiflora*	0.00	0.78	0.95	1.55	1.57	3.20	1.66	1.50	1.90
*Monodora tenuifolia*	0.00	0.00	0.50	0.00	0.00	0.00	0.00	0.00	0.00
*Piper guineense*	1.10	0.16	0.00	0.00	0.00	0.10	0.00	0.00	0.00
*Securidaca longipedunculata*	0.00	0.03	0.25	0.00	0.00	0.00	0.00	0.00	0.00
*Thonningia sanguinea*	0.00	0.03	0.05	0.55	0.00	0.00	0.00	0.00	0.00
*Uvaria chamae*	0.00	0.08	0.10	0.00	0.00	0.00	0.00	0.00	0.00
*Xylopia aethiopica*	0.50	0.00	0.00	0.00	0.00	0.00	0.00	0.00	0.00
*Zanthoxylum zanthoxyloides*	0.00	0.00	0.00	0.00	0.66	0.00	0.00	0.00	1.90

**Fig. 8:**
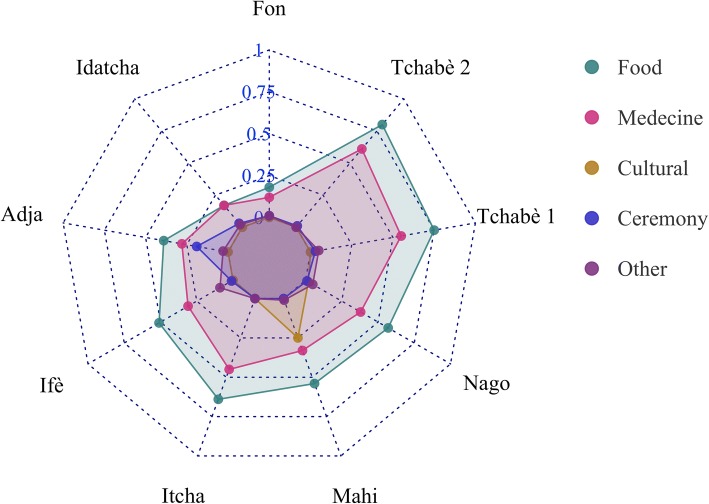
Radar plot of the use categories components of CI with respect to the sociolinguistic groups

**Fig. 9:**
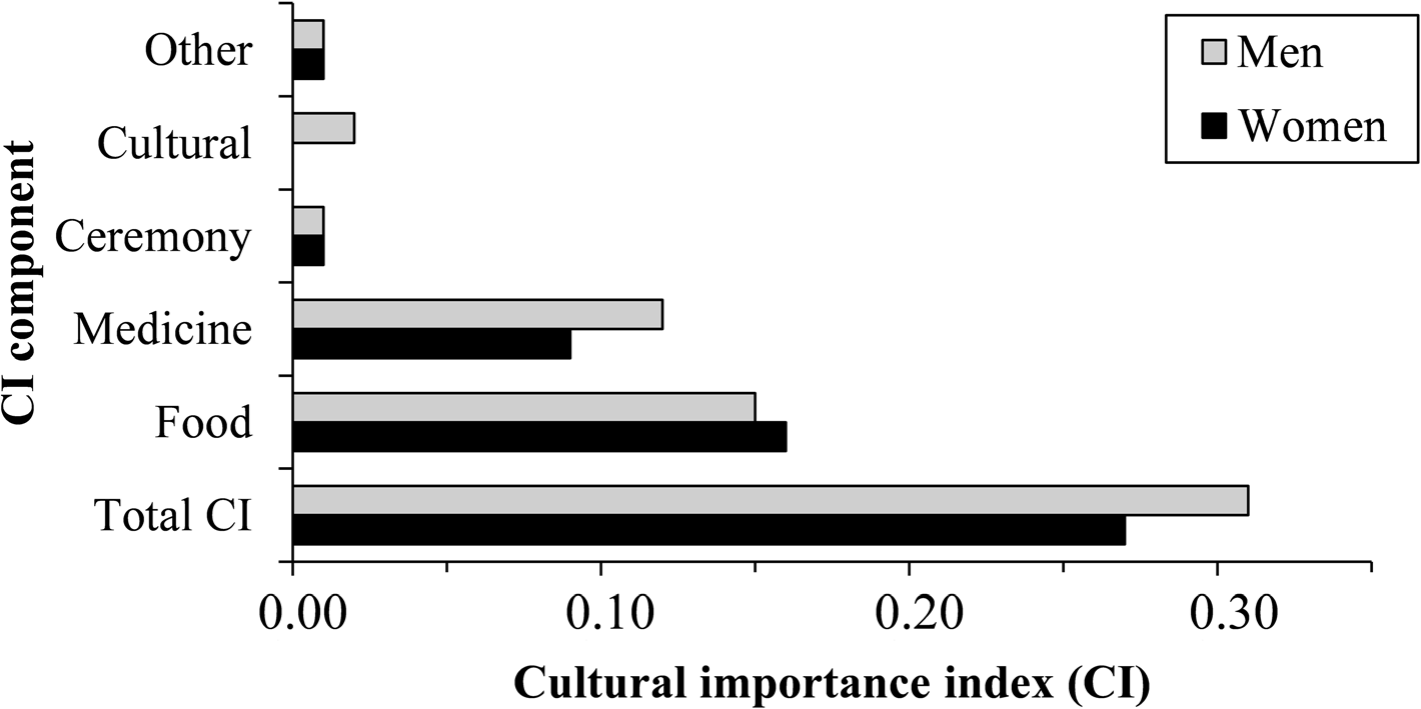
Cultural importance index (CI) of the species by gender, with the CI component of each use category

Figure 8 shows that all the sociolinguistic groups valued food and medicinal uses for the wild spices. In addition, Mahi people also made cultural use of spices (CI = 0.25) while Adja used them for ceremony (CI = 0.19) and Ifè used them for other purposes (CI = 0.09). Overall, wild spices were used for other purposes much less frequently. The most culturally important wild spices for both women and men were *L. multiflora* (CI = 1.41 and 1.44 for women and men respectively) and *A. alboviolaceum* (CI = 1.07 for women and CI = 1.49 for men). However, in comparison, men valued the wild spices more than women in an overall sense, as well as in medicine, for cultural, and other uses. By contrast, women informants used the wild spices for food purposes much more than men (Fig. 9).

Cultural importance index calculated at community and informant levels yielded different wild spices as of higher cultural significance. The most culturally important species in the surveyed communities were, in order of importance, *A. alboviolaceum*, *L. multiflora*, *A. angustifolium*, and *P. guineense* (Table 10). At informants’ level and irrespective of sociolinguistic group, gender and phytodistrict, *L. multiflora*, *A. alboviolaceum*, and *Z. zanthoxyloides* had the greater cultural importance. Therefore, *A. alboviolaceum* and *L. multiflora* appeared as the most important wild spices for the surveyed communities (CIcom = 3.00 and 2.10 respectively) and informants (CIinf = 1.28 and 1.42 respectively; Table 10).

**Table 10:** Community and informant cultural importance of the inventoried 14 wild spices

Species	CIcom	CIinf
*Aeollanthus pubescens*	0.40	0.12
*Aframomum alboviolaceum*	*3.00*	*1.28*
*Aframomum angustifolium*	*0.90*	0.19
*Aframomum melegueta*	0.40	0.08
*Clausena anisata*	0.20	0.02
*Cymbopogon giganteus*	0.30	0.02
*Lippia multiflora*	*2.10*	*1.42*
*Monodora tenuifolia*	0.30	0.05
*Piper guineense*	*0.60*	0.14
*Securidaca longipedunculata*	0.30	0.03
*Thonningia sanguinea*	0.20	0.06
*Uvaria chamae*	0.20	0.02
*Xylopia aethiopica*	0.20	0.05
*Zanthoxylum zanthoxyloides*	0.50	*0.24*

Most important species in each index are in italics

*CIcom* community cultural importance, *CIinf* informant cultural

#### Use values of the wild spices (UV)

The highest use value was reported for *Aframomum alboviolaceum* (UV = 0.93; Table 11). Fifty-three specific uses were documented for this species of which the most important was the consumption of the fresh fruit flesh by rural people (Table 12). *Lippia multiflora* had the second highest use value (UV = 0.76). The fresh young leaves constituted a well-appreciated aromatic spice across all the three phytodistricts. Leaves or inflorescences were often dried and crushed with condiments and served as seasoning in sauce. This spice was attributed stimulating effects on milk production for nursing women. *L. multiflora* was also particularly used for healing gastrointestinal ailments including indigestion, constipation, stomach-ache, dysentery, hemorrhoid, diarrhea, nausea, and vomiting. It is also said to have a great potential in regulating high blood pressure (Table 12). The species with the third highest use value was *Aframomum angustifolium* (UV = 0.18). The species was cited only in Bassila and South-Borgou phytodistricts. Its crushed or pounded fresh roots were used to relief headache (Table 12). The fruit flesh was also eaten as for *A. alboviolaceum*.

**Table 11:** Use values of the wild spice species inventoried in the Sudano-Guinean zone of Benin

Species	∑U	UV
*Aeollanthus pubescens*	18	0.08
*Aframomum alboviolaceum*	202	0.93
*Aframomum angustifolium*	40	0.18
*Aframomum melegueta*	13	0.06
*Clausena anisata*	3	0.01
*Cymbopogon giganteus*	2	0.01
*Lippia multiflora*	166	0.76
*Monodora tenuifolia*	4	0.02
*Piper guineense*	17	0.08
*Securidaca longepedunculata*	4	0.02
*Thonningia sanguinea*	13	0.06
*Uvaria chamae*	3	0.01
*Xylopia aethiopica*	8	0.04
*Zanthoxylum zanthoxyloides*	26	0.12

*U* number of quotations for a given species, *UV* use value

**Table 12:** List of wild spices, used part, processing methods, and forms and purpose of use

Use category	Plant part	Processing method	Form of use	Purpose of use	Frequency of citation		
					Ba (*n* = 61)	Sb (*n* = 59)	Zou (*n* = 93)
*Aeollanthus pubescens* Benth.							
Food	Leaves	Crush fresh leaves and mix with seasoning and sesame	Eat as sauce	Human nutrition	–	17	–
				Milk production stimulation for nursing women	–	1	–
				Diarrhea	–	2	–
				Intestinal worms	–	1	–
	Above ground	Crush and mix with seasoning and sesame	Eat as sauce	Stop hemorrhage for nursing women	–	2	–
Medicine	Leaves	Boil in water as decoction	Drink the liquid 3 times/day	Fever	–	2	–
Other		Pound the fresh leaves and mix with traditional soap *koto*	Take a shower with	Deodorant for nursing women	–	1	–
*Aframomum alboviolaceum* (Ridley) K.Schum.							
Food	Leaves	Boil young leaves and mix with seasoning	Eat as vegetable	Human nutrition			1
	Fruits	Remove the cockleshell	Eat the pulp	Human nutrition	57	53	86
				Malaria	1	5	1
				Cough	–	1	–
		Crushed dried cockleshells as condiment in sauce	Eat	Human nutrition	2	–	–
	Root	Crushed roots as aromatic spice in sauce	Eat as sauce, contra-indicated with pregnant women	Milk production stimulation for nursing women	3	–	–
Medicine	Leaves	Boil leaves with stem and roots in water as decoction	Drink the liquid and take a shower 3 times/day	Fever and malaria	5	3	6
			Drink the liquid 2 times/day	Stomach-ache	–	1	–
				Milk production stimulation for nursing women	1	–	–
		Soak dried leaves in water for 2–3 days	Drink the liquid and take a shower 3 times/day	Fever and malaria	1	–	–
		Boil leaves in water as decoction	Take a shower	Fortify infants	2	–	–
			Drink the liquid, a glass for adult, half for children	Dermatosis	1	–	1
		Boil in water with *Caesalpinia pulcherrima* leaves, then add a piece of sugar	Drink a small glass 2 times/day	Icterus and yellow-fever	2	–	–
		Triturate the leaves	Drink the juice 2 times/day	Hemorrhoid	–	–	1
		Boil dried leaves in water as decoction. Add 2–3 pieces of sugar	Drink the liquid 4–5 times/day	Anemia for children	1	–	–
		Boil in fermented corn water the yellowed leaves with *A. angustifolium* fruits plus tough potash	Drink a glass/day	Sexual weakness	1	–	–
		Boil in the fermented corn water yellowed leaves with cockleshells of *A. angustifolium* fruits and tough potash	Drink the liquid 2 times/day	Painful menstruation	1	–	–
		Fresh leaves	Rub the leaves juice against the body	Stop itching or insect bite	–	1	–
	Fruits	Remove the cockleshell and Soak the pulp including the seeds in water for 2–3 h	Drink the liquid	Malaria	2	3	3
				Hematuria	–	1	–
		Pound dried cockleshells with dried seeds of *A. melegueta*	Make 9 scarifications at the hip	Hip-ache	–	–	1
		Remove the cockleshells	Smell the scent or eat the pulp	Nausea for pregnant women	2	–	–
		Boil in water as decoction	Drink the liquid once the day	Fortify infants	–	–	1
		Boil 4 fruits with other ingredients as decoction	Drink the liquid 3 times/day	Infertility for women	–	–	1
		Pound fresh fruits and make small bowls	Insert the bowls in the vagina before sleeping	Absence of menstruation	1	–	–
		Soak in water with roots	Drink the liquid 3 times/day	Strengthening of bones	2	–	–
	Root	Wash fresh roots	Eat raw and swallow the juice	Snake bite	–	–	1
				Stomach-ache	–	–	1
		Pounded fresh roots mix with other ingredients	Make a poultice	Snake bite	1	–	3
		Soak in Sodabi or in sorghum fermented beverage for 2–3 days	Drink a small glass 2 times/day - contra-indicated with pregnant women	Stomach-ache	1	–	–
		Soak with 7–8 *M. myristica* seeds and 4 *Garcinia kola* seeds	Drink the liquid 3 times/day	Sexual weakness	–	–	1
		Dried roots and dried fruits plus tough potash crushed: mixed the powder palm almonds oil	Eat the mixture, on the morning after eating something sweetened	Intestinal worms	–	1	–
		Crush dried roots with tough potash as powder	Lap 3 times/day	Intestinal worms	–	–	1
		Boil in water with roots of *Imperata cylindrica*	Drink the liquid 3 times/day	Tuberculosis	1	–	–
		Boil in water as decoction	Rinse the mouth	Tooth decay	1	–	–
			Take a shower	Fever	–	1	–
			Rub the liquid against the wound	Wound healing	1	–	–
			Drink the liquid 3 times/day - contra-indicated with pregnant women	Malaria	1	–	–
		Crushed with *M. myristica* seeds, warm	Rub the mixture against the body	Swelling and edema	1	–	–
		roast with *Imperata cylindrica* roots, 10 pods of *M. myristica*, 10 *Garcinia kola* seeds and tough potash as powder	Mix with porridge, once on the morning	Infertility for women	–	1	–
		Soak in water for 4 days	Drink the liquid and take a shower 2 times/day	Fortify the body	–	1	–
		Cut and Soak in the water of beverage	Drink	Poultry disease healing	–	1	–
	Stem	Cut the stem and add 3 seeds of *A. melegueta*	Eat and swallow the juice	Stomach-ache	–	–	1
		Boil in water as decoction, add shea butter	Drink the liquid	Cough	–	1	–
		Boil in water as decoction	Drink the liquid 3 times/day	Miscarriage	1	–	–
	Seeds	Macerate in the traditional palm alcohol (sodabi) dried seeds with papaya’s dried seeds	Drink a small glass 3 times/day	Stomach-ache	1	–	–
		Take some seeds and add with a *Garcinia kola* seed	Eat	Cough	1	–	–
		roast dried seeds as powder and mix with porridge	Drink the porridge	Vertigo	–	–	–
Other	Leaves	Triturate the leaves in water	Take a shower	Bad body smells	–	1	–
		Fresh leaves	Wrap up maize based paste (akassa)	Trade	–	–	5
	Stem	Fresh stems	As string to attach luggage on the field	Home use	–	6	–
			As wipe to punish children	Home use	–	5	–
Ceremony	Seeds	Dried seeds	Eaten by fetishist to activate incantations	Magico-religious	–	2	–
*Aframomum angustifolium* (Sonn.) K.Schum.							
Food	Leaves	Crushed leaves as aromatic spice in sesame sauce	Eat the sauce	Human nutrition	2	–	–
				Stop hemorrhage for nursing women	–	1	–
	Fruits	Remove the cockleshell	Eat the pulp	Human nutrition	5	–	–
	Root	Crushed roots as aromatic spice in sauce	Eat as sauce	Human nutrition	1	–	–
				Fortify nursing women	1	–	–
				Icterus	1	–	–
				Headache	1	–	–
Medicine	Leaves	Crushed leaves mixed with salt or in sauce	Drink the mixture or eat the sauce	Stomach-ache	1	–	–
		Soak fresh leaves in water	Rinse the face 3 times/day	Swelling of the face	–	1	–
		Fresh leaves	Rub the leaves juice against the face 3 times/day	Swelling of the face	–	1	–
		Leaves, roots and fruits as key ingredients for the recipe	Drink	Discard snakes and snake bite healing	1	–	–
	Fruits	Remove the cockleshell and Soak the pulp including the seeds in water	Drink the liquid	Malaria	1	–	–
				Hematuria	–	1	–
		Crush or pound and mix with shea butter	Rub the mixture against the body	Ache	1	–	–
		Soak in water with roots	Drink the liquid 3 times/day	Strengthening of bones	1	–	–
	Root	Boil in water as decoction with potash	Drink the liquid	Anemia	–	1	–
		Crush or pound fresh roots	Rub the mixture against the body	Swelling and edema	1	2	–
		Crush or pound fresh roots	Rub the mixture against the head	Headache	7	–	–
		Boil in water as decoction	Take a shower	Ache	–	1	–
				Fever	–	1	–
		Crush or pound fresh roots	Put a little in the nostrils	Cold	1	–	–
		Crush fresh roots	Put the paste obtained on the decayed tooth	Tooth decay	–	1	–
		Soak in water for 5 min the crushed fresh roots	Rub against the head and rinse the face	Vertigo	–	1	–
		Soak in water with fruits peduncle and leave under the sun	Take a shower	Measles	1	–	–
	Stem	Soak in germinated corn beverage	Rinse the face	Swelling of the face	–	1	–
		Cut the fresh stems	Eat and swallow the sap	Cough	1	–	–
Other	Stem	Fresh stems	As string to attach luggage on the field	Home use	–	2	–
			As wipe to punish children	Home use	–	1	–
Ceremony	Seeds	Dried seeds	Eaten by fetishist to activate incantations	Magico-religious	1	1	–
*Aframomum melegueta* (Roscoe)							
Medicine	Fruits	Remove the cockleshell and Soak the pulp including the seeds in water	Drink the liquid	Malaria	1	–	–
				Hematuria	–	1	–
		Crush or pound and mix with shea butter	Rub the mixture against the body	Ache	1	–	–
		Soak in water with roots	Drink the liquid 3 times/day	Strengthening of bones	1	–	–
	Root	Sock in palm wine	Drink the liquid for 9 days (men) or 7 day (women)	Asthenia	–	–	1
	Seeds	Crush dried seeds and mix with water	Drink the liquid and drip on the eyes, contra-indicated with pregnant women	Anemia	–	–	1
			Drink the liquid 2–3 times/day, contra-indicated with pregnant women	Diarrhea	–	–	1
		Crush dried seeds and mix with palm alcohol	Drink the liquid 2 times/day, contra-indicated with pregnant women	Stomach-ache	–	–	1
		Soak with *X. aethiopica* fruits	Drink a small glass 2 times/day, contra-indicated with pregnant women	Stomach-ache	–	–	1
		Crush dried seeds as powder	Make scarifications for 9 days (men) or 7 days (women)	Paralysis	–	–	1
		Soak in palm alcohol with *P. guineense* and *X. aethiopica* fruits, ginger and lemon	Drink the liquid 3 times/day, contra-indicated with pregnant women	Ulcer	–	–	1
Ceremony	Seeds	Dried seeds	Eaten by fetishist to activate incantations	Magico-religious	–	–	10
*Clausena anisata* (WilId.) Hook.f. ex Benth.							
Medicine	Leaves	Boil in water as decoction	Take a shower	Dermatosis	–	–	1
	Root	Boil in water as decoction	Drink the liquid 2 times/day	Hemorrhoid	–	–	1
Ceremony	Leaves	Fresh leaves	Make magic to counter tchakatou dark magic	Magico-religious	–	–	1
*Cymbopogon giganteus* (Hochst.) Chiov.							
Food	Leaves	Attach the leaves and use as aromatic in *Cleome gynandra* leaves sauce	Eat the sauce	Human nutrition	–	–	2
*Lippia multiflora* Moldenke							
Food	Leaves	Crush the young shoots and mix with seasoning and crushed sesame or peanut as aromatic spices	Eat the sauce	Human nutrition	49	54	29
		Boil in water and mix with seasoning as vegetable sauce			1	–	2
		Dry and reduce in powder	Sprinkle the sauce	Human nutrition	43	35	17
		Boil fresh leaves in water with corn	Eat	Human nutrition	–	2	2
		Soak in water with corn and grind	Eat as akassa	Human nutrition	–	–	1
		Infuse dried leaves in warm water as aromatic tea	Drink the tea	Human nutrition	1	1	–
	Flower	Dried flowers in whole or crushed	Sprinkle the sauce	Human nutrition	44	35	17
				Milk production stimulation for nursing women	2	1	–
				Healing uterine wounds for nursing women	3	1	–
				Indigestion, constipation and stomach-ache	8	5	–
				Dysentery and Hemorrhoid	2	1	–
		Boil flower or leaves in water with seasoning as spice for spicy meat	Eat	Human nutrition	–	1	5
Medicine	Leaves	Boil in water as decoction	Drink the liquid and take a shower 3 times/day	Fever and malaria	6	9	25
				Fortify infants	2	5	6
				constipation and Stomach-ache	1	1	7
				Diarrhea	–	3	–
				Measles	1	1	–
			Cover the head and take a steam bath	Headache	2	–	–
			Rinse the eyes	Sore eyes	–	–	1
		Boil in water as decoction or Soak in alcohol	Drink the liquid	Blood-pressure	3	–	–
		Boil in water with stem and roots as decoction		Cold and cough	2	–	2
				Dysentery and Hemorrhoid	–	1	2
			Drink the liquid and rinse the body or take a shower 2 times/day	Dermatosis and wound healing	–	2	5
		Boil in water leaves and roots	Drink the liquid	Healing uterine wounds for nursing women	4	–	–
				Nausea and vomiting	3	1	–
		Triturate the leaves	Inhale the gas and drip on the eyes	Headache	–	–	1
			Put the juice into the nostrils	Cold	–	–	1
			Drink the juice before going to hospital	Snake bite	1	–	–
		Fresh leaves	Eat and swallow the juice	Cough	–	–	–
	Root	Boil in water as decoction	Drink the liquid 2–3 times/day	Anemia for children	1	–	1
				Nausea and vomiting	10	–	–
				Indigestion and Stomach-ache	2	9	–
				Diarrhea	–	1	–
				Ulcer	–	2	–
				Intestinal worms	2	–	–
			Drink the liquid and take a shower	Fever and malaria	–	12	2
				Fortify infants	–	3	–
		Boil in water as decoction with small red onions, *X. aethiopica* fruits and *M. myristica* seeds	Drink the liquid and take a shower	Dermatosis and wound healing	–	7	1
		Crush or pound fresh roots	Rub the mixture against the body	Swelling and edema	1	–	1
		Soak in water for 2 h	Drink the liquid 3 times/day	Painful menstruation	1	–	–
		Soak in water for 3 days with *X. aethiopica* fruits	Rinse the eyes	Sore eyes	–	1	–
		Crush dried roots with 9 *A. melegueta* seeds and *X. aethiopica* fruit as powder	Inhale the powder	Headache	1	–	–
	Stem	Boil in water as decoction	Drink the liquid 3 times/day	Asthma	–	1	–
Cultural	Flower	Dried flowers	Use to spicy sauce	Food	–	21	–
*Monodora tenuifolia* Benth.							
Food	Seeds	Roast the seed to remove the cockleshells and crush the almond, use as aromatic spice in sauce	Eat the sauce	Human nutrition	–	–	1
		Crush almonds with *X. aethiopica* fruits and mix with pepper, chili, other spices and seasoning	Eat as sauce	Stomach-ache	–	–	1
Medicine	Seeds	Boil in water with *X. aethiopica* fruits, *U. chamae* roots and small red onions	Drink the liquid 3 times/day	Stomach-ache	–	–	1
				Black menstruations and absence of menstruations	–	–	1
		Crush the almond and mix with palm almond oil	Rub the mixture against the body	Fever	–	–	1
		Boil in water as decoction	Take a shower	Fever	–	–	1
	Flower	Char flowers with *U. chamae* roots as powder and an *A. melegueta* fruit pod	Sprinkle wounds	Incurable wounds healing	–	–	1
	Bark	Crush dried bark and 7 *A. melegueta* seeds as powder and mix with palm almond oil	Put the mixture in the wounds	Wound healing	–	–	1
		Soak fresh barks in the water	Rinse a wound	Wound healing	–	–	1
	Root	Pound dried roots and mix with sugar and sulfur as powder	Mix the powder with the porridge	Dysentery and Hemorrhoid	–	–	1
Ceremony	Seeds	Dried seeds	Used by fetishist for various ceremonies	Magico-religious	–	–	4
*Piper guineense* Schumach. & Thonn.							
Food	Leaves	Fresh leaves crushed or in whole as aromatic spice in sauce	Eat	Human nutrition	–	–	13
	Fruits	Crush dried fruits and mix with seasoning			–	–	11
	Stem	Cut fresh stems and put in the sauce			–	–	2
	Root	Put fresh roots in the sauce			–	–	1
Medicine	Leaves	Boil in water as decoction	Drink the liquid	Dermatosis	–	–	1
	Fruits	Sock in water dried fruits with ginger and fruit of *X. aethiopica*	Drink the liquid, contra-indicated with pregnant women	Dermatosis	–	–	1
		Soak in alcohol dried fruits with *A. melegueta* and *X. aethiopica* fruits		Ulcer	–	–	1
				Hematuria	–	–	1
		Soak dried fruits in alcohol	Drink the liquid 2 times/day	Blood-pressure	–	–	1
		Crush dried fruits and mix with salt	Drink the mixture		–	–	–
Ceremony	Fruits	Dried fruits	Used by fetishist for ceremonies of fâ	Magico-religious	–	–	5
*Securidaca longipedunculata* Fresen.							
Medicine	Root	Crush or pound the roots	Make a poultice	Edema and abscess	–	–	2
		Soak in water with *X. aethiopica* fruits and *M. myristica* seeds	Drink the liquid	antibiotic	–	–	1
		Boil in water with *U. chamae* and *Z. zanthoxyloides* roots, *X. aethiopica* fruits, *M. myristica* seeds and small red onions	Drink the liquid once the day	Stomach-ache	–	–	1
		Soak in alcohol with garlic and *A. melegueta* seeds	Drink the liquid	Fever	–	–	1
*Thonningia sanguinea* Vahl							
Medicine	Root	Rub against a rock	Rub the root juice against the body	Edema, abscess, swelling, wound and mumps	10	–	1
		Dry and reduce in powder the roots with papaya roots and fruits of *A. melegueta*		Hemorrhoid	1	–	–
		Crush the roots	Rub the past against the body	Guinea-worm disease	–	–	1
*Uvaria chamae* P.Beauv.							
Food	Fruits	Ripe fruit	Eat	Human nutrition	–	–	1
	Root	Crush and mix with seasoning as aromatic spice	Eat	Human nutrition	–	–	1
Medicine	Leaves	Boil in water as decoction	Take a shower	Fever	–	–	1
	Root	Soak in alcohol with *X. aethiopica* fruits, *M. myristica* and *A. melegueta* seeds	Drink the liquid 2 times/day	Stomach-ache	–	–	2
		Boil in water with ripe palm fruits	Drink the liquid	Anemia	–	–	1
*Xylopia aethiopica* (Dunal) A.Rich.							
Medicine	Fruits	Boil or soak in water with *A. melegueta* seeds	Drink the liquid 3 times/day	Stomach-ache	–	–	3
		Dried fruits	Eat the fruit and swallow the juice	Nausea and vomiting	–	–	1
		Boil in water with other ingredients as decoction	Drink the liquid	Infertility for women	–	–	2
		Boil in water as decoction	Drink the liquid	Fortify infants	–	–	1
		Crush dried fruits and mix with alcohol	Rub the mixture against the body	Edema, swelling and dermatosis	–	–	2
		Crush dried fruits as powder and mix with sugar	Mix the powder with the beverage and drink	Asthenia	–	–	1
Ceremony	Fruits	Dried fruits	Used by fetishist for various ceremonies	Magico-religious	–	–	2
*Zanthoxylum zanthoxyloides* (Lam.) Zepernick & Timler							
Food	Root	Remove, dry and crush roots’ barks as powder	Spicy sauces and eat, contra-indica with pregnant women	Human nutrition	25	–	–
				Milk production stimulation for nursing women	9	–	–
				Stomach-ache and ulcer	4	–	–
				Dysentery and diarrhea	2	–	–
				Healing uterine wounds for nursing women	5	–	–
Medicine	Root	Soak the roots’ barks in alcohol	Drink the liquid 2–3 times/day, contra-indicated with pregnant women	Stomach-ache	14	–	–
				Nausea and vomiting	1	–	–
				Intestinal worms	1	–	–
				Sexual weakness	1	–	–
		Soak the roots’ barks in alcohol with *A. melegueta* seeds		Abortion	2	–	–
		Soak the roots’ barks in alcohol with garlic, pepper, chili, *X. aethiopica* fruits, *M. myristica* and *A. melegueta* seeds	Drink the liquid 2–3 times/day, contra-indicated with pregnant women	Ulcer	3	–	–
				Menstruation issue	1	–	–
		Boil in water the roots’ barks with garlic, pepper, chili, *X. aethiopica* fruits, *M. myristica* and *A. melegueta* seeds	Drink the liquid 2–3 times/day, contra-indicated with pregnant women	Sexually transmissible infections	1	–	–
		Boil in water the roots’ barks with ginger leaves as decoction	Drink the liquid	Painful and dark menstruation	2	–	–

*Ba* Bassila, *Sb* South-Borgou

#### Uses diversity

**Fig. 10:**
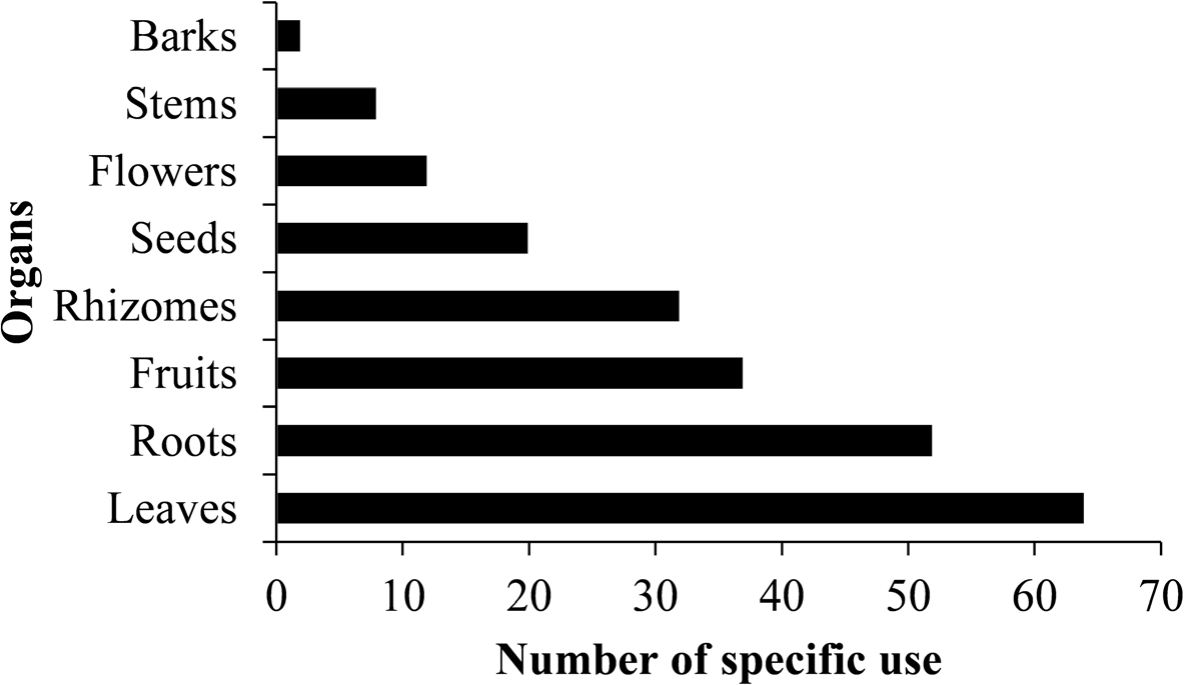
Number of specific use for each plant part of wild spice in the Sudano-Guinean Zone

A total of 205 specific uses were recorded for the 14 wild spices in the three phytodistricts. The first three species with the highest specific uses were *A. alboviolaceum* (53 uses), followed by *L. multiflora* (45 uses) and *A. angustifolium* (29 uses), while only one specific use was mentioned for *Cymbopogon giganteus*. Wild spices were used mostly for medicine (144 uses) and lesser for cultural (1 use) purposes. All uses are documented in Table 12. Leaves had the highest number (64 uses) of specific uses while the bark (2 uses) had the lowest specific uses (Fig. 10).

#### Local perception of the dynamic of wild spices populations and threatening factors

**Fig. 11:**
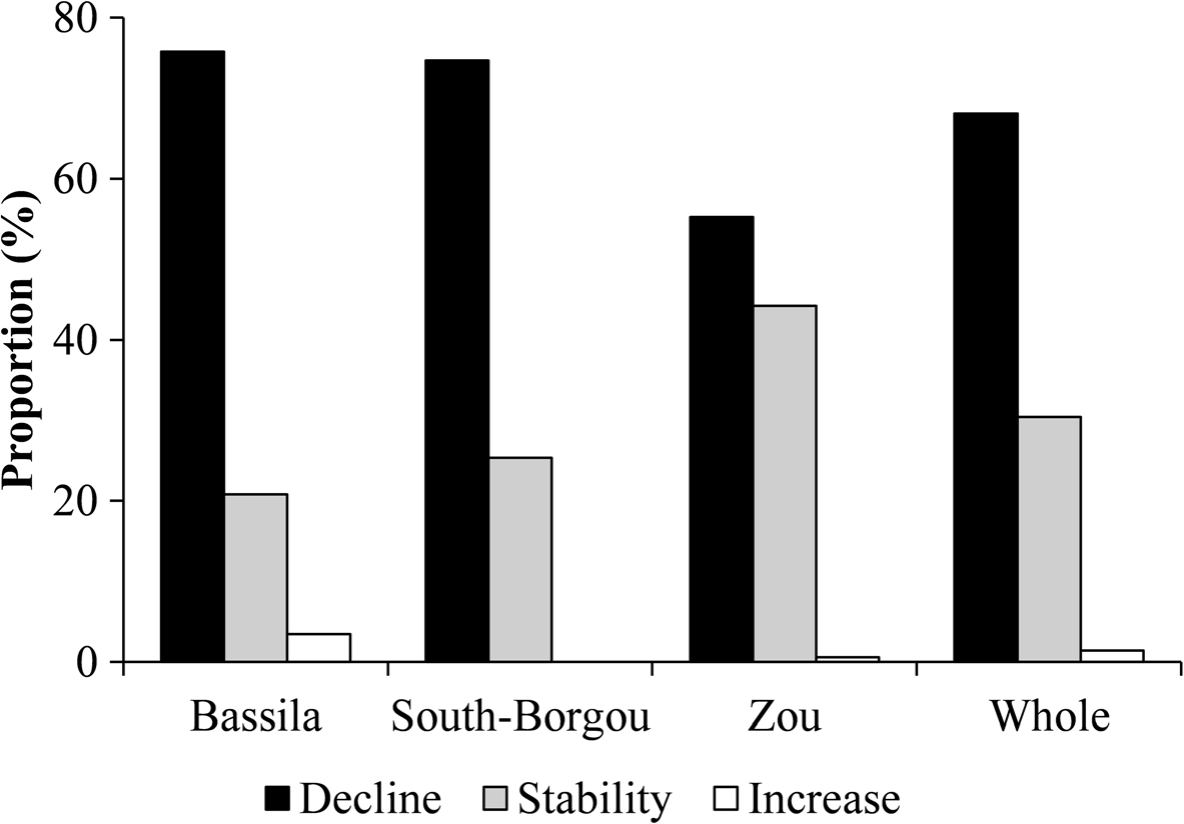
Local perception of the dynamic of wild spice populations by phytodistrict

Most of the informants (92.02%) indicated their perception regarding the dynamic of wild spices’ populations. Perceptions varied significantly among phytodistricts (*p* < 0.001), with no variation as regards age categories (*p* = 0.063) and gender (*p* = 0.131). Most respondents indicated that wild spices’ populations declined over time. Stability was mentioned mostly in the phytodistrict of Zou; meanwhile, an increase was mentioned mainly in Bassila. No increase in the dynamic was recorded in the South-Borgou phytodistrict (Fig. 11).

**Fig. 12:**
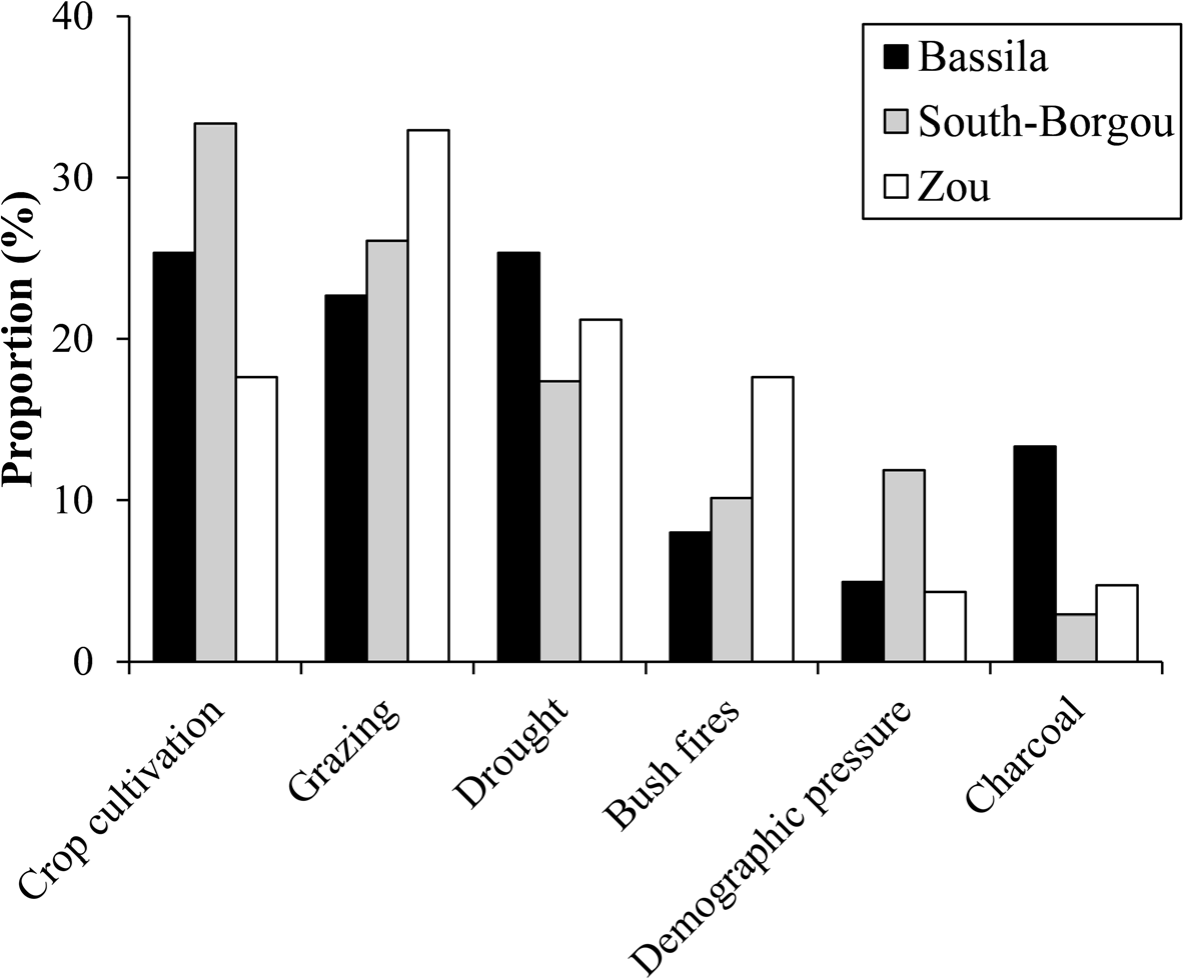
Factors purported responsible of the decline of the wild spice populations in the Sudano-Guinean zone

The main threatening factors purported responsible for the decline in wild spices’ populations were agriculture, grazing, and drought in the three regions (Fig. 12). The most important factor was agriculture in South-Borgou (cited by 33.33% of respondents), grazing in Zou (32.94%), whereas drought and agriculture were equally quoted in Bassila (25.33% each). Bushfires, demographic pressure, and charcoal production also contributed to a lesser extent to the decline of the spices population in the Zou, South-Borgou, and Bassila phytodistricts respectively.

#### Local perception of the nutritional value of the wild spices

**Fig. 13:**
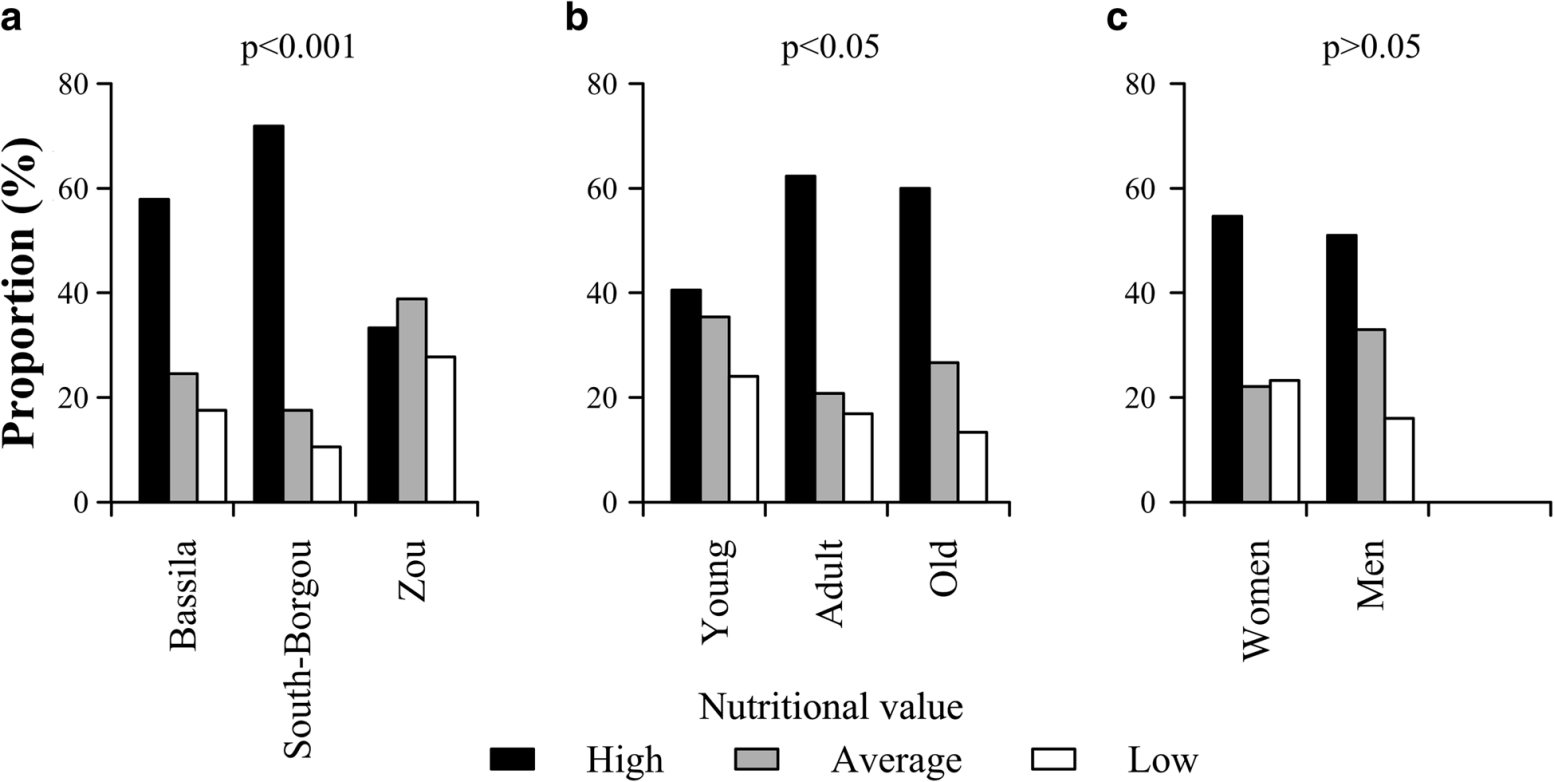
Local perceptions of the nutritional value of the wild spices. **a** Phytodistrict. **b** Age category. **c** Gender. *p* = *p* value from Fisher’s exact text

Overall, 87.32% of informants indicated their perception of the nutritional value of the wild spices that they use in their diet. Significant variation of perceptions exists among phytodistricts (*p* < 0.001) and age categories (*p* = 0.038). However, no significant variation was observed between gender (*p* = 0.219). High nutritional value was mentioned mostly in South-Borgou and Bassila phytodistricts (respectively 71.93% and 57.89% of the respondents) whereas people from Zou (38.89% of respondents) assigned an average nutritional value to the wild spices. Adults (62.34%) and old informants (60%) acknowledged higher nutritious value to wild spices compared to young (40.51%). Overall, women and men perceived wild spices as highly nutritious (Fig. 13).

### Priority wild spices for conservation

The Point Scoring Procedure (PSP) method yielded a list of priority wild spices for conservation, with *M. tenuifolia*, *L. multiflora*, *X. aethiopica*, *S. longipedunculata*, and *A. melegueta* as top five highest priority. For the PSPW method, *M. tenuifolia*, *X. aethiopica*, *L. multiflora*, *Z. zanthoxyloides*, and *S. longipedunculata* appeared as the top five highest priority. For the CRS method, the top five priority wild spices were *M. tenuifolia*, *X. aethiopica*, *L. multiflora*, *A. melegueta*, *Z. zanthoxyloides*, and *A. angustifolium*. For the BRS method, the top five priority species were *A. melegueta*, *M. tenuifolia*, *P. guineense*, *U. chamae*, and *X. aethiopica*. By crossing results of each method, the top five species for conservation, selected in the Sudano-Guinean zone, were *A. alboviolaceum*, *L. multiflora*, *X. aethiopica*, *Z. zanthoxyloides*, and *M. tenuifolia* (Table 13).

**Table 13:** List of top 5 priority wild spices for conservation in the Sudano-Guinean Zone in Benin

Species	PSP	PSPW	CRS	BRS	Total
*Aframomum alboviolaceum*	x	x	x	x	4
*Lippia multiflora*	x	x	x	x	4
*Xylopia aethiopica*	x	x	x	x	4
*Zanthoxylum zanthoxyloides*	x	x	x	x	4
*Monodora tenuifolia*	x	x	x	x	4

*PSP* point scoring procedure, *PSPW* point scoring procedure with weighing, *CRS* compound ranking system, *BRS* binomial ranking system

## Discussion

### Diversity of the wild spices across phytodistricts

A total of 14 wild spices was recorded throughout the Sudano-Guinean zone. This species richness seems relatively low when compared to that reported from a similar study conducted in the Niger Delta area in Nigeria (24 species) [52]. This difference could come from the location of part of Benin in the Dahomey-Gap, an approximately 200-km-wide savanna corridor from Ghana to Benin, separating the Upper and the Lower Guinean rain forest blocks [53]. This phenomenon does not allow an impressive biological diversity but rather induced a relatively few number of endemic species to Benin as most species are shared with neighboring countries. This situation added to NTFPs’ overexploitation, climate change, and raging demography with its subsequent consequences, increasingly compromises indigenous species persistence, thus, leading to the scarcity or disappearance of critical species [8, 54]. Further, extensive land use and cover transitions and the intensification of human pressure in the surrounding of protected areas increases the vulnerability of species [55]. Consequently, the wild spices should be conserved through their integration in the traditional agroforestry systems.

Wild species used as spices in the Sudano-Guinean zone belong mainly to the families of Zingiberaceae, Annonaceae, and Rutaceae which are well-known for holding much of aromatic plants and spices [56]. The inventoried wild spices richness was predominated by Afrotropical and Guineo-Congolian species and a low representation of Sudano-Guinean species. This could be explained by human migration associated with seed dispersal and propagation of the species across areas, ruling out isolation by distance, as reported for *Caesalpinia bonduc* L. [57]. Likewise, the genetic diversity and genetic differentiation might be low for these species, due to parental links between populations. This may reduce their ability to survive and adapt to changing environmental conditions.

### Traditional knowledge, informant consensus, cultural importance and use value of the wild spices

The study showed how Traditional knowledge (TK) of wild spices is structured in the Sudano-Guinean Zone. Our results support the general hypothesis that TK depends on geographical location, sociolinguistic group, and gender [58]. Indeed, people in Bassila phytodistrict held the greatest level of knowledge, and regarding the sociolinguistic difference in TK, Tchabè informants from Bassila reported more uses than other sociolinguistic groups. This suggests that the wild spices are mostly used in the Bassila phytodistrict and less in the Zou and South-Borgou phytodistricts. Male informants reported more uses than female ones. Although this finding does not confirm the general statement that women are reservoirs of traditional plant knowledge [59], similar findings have been reported in Benin [60, 61] and Mexico [62]. As suggested by Voeks [59], this might be due to gender division of the space and labor.

The most culturally important wild spices as revealed from our data were, by order of importance, *L. multiflora*, *A. alboviolaceum*, and *Z. zanthoxyloides*. People in Bassila phytodistrict culturally valued more wild spices than those from the other phytodistricts. This is congruent with the TK pattern of wild spices. The two species with the highest cultural importance (CI) value were the most quoted in all three phytodistricts. This similarity may have resulted from a common cultural background. Irrespective of gender and age category, studied sociolinguistic groups valued different wild spices. For instance, *A. alboviolaceum* was most culturally important for Fon, *L. multiflora* for Mahi, and *Z. zanthoxyloides* for Tchabè from Bassila. Such intercultural variation in wild plant species use is consistent with previous studies in Benin [19, 63, 64]. Irrespective of sociolinguistic group, gender and age categories, the most culturally important uses of the wild spices were for food, medicine and cultural practices respectively. This finding clearly attests that spices are initially intended for food. With respect to gender, the most culturally important use for men was medicinal and to some extent ceremonial, while women, almost exclusively, were specialized in using the spices for food. The medicinal and ceremonial relatedness of preference use values for men could be associated to the cultural custodianship of men in the sociolinguistic groups studied. In most villages in Benin, men are assumed to be the tradition guardian and are often engaged in ceremonial and magico-religious activities. This makes them more knowledgeable in these use categories. For instance, *Monodora tenuifolia*, a wild spice used by Mahi men for rituals in convents of traditional divinities, is totally unknown to women of the same sociolinguistic group. Similarly, African black pepper (*Piper guineense*) fruits constitute an essential component in Fâ rituals [65] and are often harvested from the sacred groves of Badjamè by Adja priests. Meanwhile, Adja women harvested the leaves of the same plant in surrounding of the sacred groves for flavoring soups but none of them knew that the plant they always harvest was actually black pepper plant. As all these roles are generally devoted to men, they may likely have more knowledge of medicinal and ceremonial plants than women. On the other side, women are most involved in performing household labors and would thus be more knowledgeable on food plants and less on ritual or ceremonial plants. The difference of preference of use between men and women confirms the hypothesis of gender division of space and labor within households [59].

Overall, the informant consensus analysis revealed a high level of agreement among the informants for all use categories indicating a more consistent use of wild spices. This opens an avenue for the sustainable management of these neglected resources through promotion of their domestication involving local communities. As it could be expected, the highest degree of agreement was found for food use category, and followed by medicinal uses. This may be due to the high number of use reports for only ten wild spices consumed in the study area (Table 7). It indicates that information pertaining to the use of food/edible wild spices is shared among informants in the surveyed communities. More interestingly, men and women shared the same level of agreement suggesting that female informants are as well informed as male informants. Moreover, the degree of consensus varied among the sociolinguistic groups with Idatcha people having the least agreement (*F*_ic_ = 0.88). It appeared thus that these people collect randomly wild spices for particular uses contrary to Tchabè from Zou phytodistrict who relied on only two wild spices for all uses (*F*_ic_ = 0.98). Therefore, the rarefaction or loss of one of the many wild spices available for Idatcha is expected to have a little impact on the overall use and the traditional practices in this sociolinguistic group [66]. Meanwhile, more attention should be devoted to the two species used by Tchabè in the Zou phytodistrict [67].

The wild spices with high use values were *A. alboviolaceum*, *L. multiflora*, *A. angustifolium*, and *Z. zanthoxyloides* respectively. The highest UV of *A. alboviolaceum* comes from the fact that the species is a multipurpose plant widespread in savannas across Benin and most farmers use it as wild food and thirst quencher in the field which are often far from the villages. Moreover, many farmers value its stem as string to make bundle of firewood and bind luggage a (Table 12). This emphasizes the key role and value of wild foods in the daily life of agricultural communities [68].

Two of the spices (*L. multiflora* and *Z. zanthoxyloides*) were used in the treatment of gastrointestinal diseases, ailments which are common in the study area due to low availability of drinking water and sanitation facilities [69]. In addition to their use as food spice, *Z. zanthoxyloides* and *L. multiflora* were also attributed stimulating effects on milk production in nursing women. This corroborates previous findings of Segnon and Achigan-Dako [70] and Atanasso et al. [71] who reported the same uses for *Z. zanthoxyloides* and *L. multiflora* respectively.

Considering wild spices use categories, the high number of specific uses (144 uses) as medicine compared to food category (39 uses) contrasts however with the previous use pattern of wild spices: food use category remained first. Nevertheless, this finding is congruent with the statement that indigenous people value wild spices more for their ethnomedicinal uses than for spicing food [52]. Another reason might result from intercultural knowledge gaps in the use of some species. For instance, Fon people did not make food use of *L. multiflora* at all, while the species was highly culturally important for Mahi for food needs. Since home gardens are critical in conserving plant biodiversity, their promotion as home for wild spices should be encouraged, particularly in areas of Bassila occupied by Tchabè. This will help to diversify household incomes, conserve the species, and strengthen the food and nutritional security of rural people.

Most of the respondents acknowledged decline of wild spice populations over the time. Main factors responsible for this decline as perceived by local people were both anthropogenic and natural. Among anthropogenic factors, crop cultivation stood first followed by grazing. This finding is in accordance with a previous study on *Sclerocarya birrea* where land clearance for agricultural purposes, carving, and drought were the main threatening factors [72].

### Priority wild spices for conservation

In recent years, increasing attention has been given to the prioritization of wild plant species and their conservation in Benin. While the previous prioritization studies undertaken so far have targeted different groups of species such as non-timber forest products (NTFPs) [64], wild edible plants [20], crop wild relatives [47], neglected and underutilized species [73], and timber species, none of these studies has focused on wild spices. The only previous study that listed some wild spices for conservation is the Red List of threatened plant species of Benin [4], with six species (*Monodora myristica*, *Tetrapleura tetraptera*, *Xylopia aethiopica*, *X. rubescens*, *Zanthoxylum gilletii*, and *Z. zanthoxyloides*) documented as threatened based on the IUCN criteria [74]. Four of those wild spice taxa were missing from our inventory and the missing taxa included two species (*M. myristica* and *Z. gilletii*) whose respective ecological range was out of the extent of the study area, and two others that were not recorded from our ethnobotanical inventory. This could also suggest that threat is a temporally dynamic process influenced by several external (human-associated and environment-related) and internal (reproduction, viability, inbreeding, genetic erosion, and adaptability) factors [75], and a species that has a low conservation concern today can become highly threatened in the future [76]. Urgent conservation action to safeguard the wild spices would be the only way to ensure the availability of these resources for the future generations. Indeed, agriculture in Benin and in sub-Saharan Africa in general is characterized by rainfed and low-input subsistence farming practiced by rural households [77], and more new lands are cleared every year for food production, as most soils have low fertility. A step-change in current agricultural system is hence needed if the impact of farming on wild plant species is to be mitigated [78]. However, this does not mean that food production must be neglected *ipso facto* for the conservation of wild plant species. Rather, policymakers should find a trade-off between agriculture and conservation in order to feed the fast-growing populations while preserving wild resources for future generations. For instance, sustainable intensification of agriculture would be an attractive alternative that can reduce considerably agriculture contribution to habitat destruction [79], and increase sustainability and effectivity of conservation actions targeting wild spices.

Although the method and criteria used were not the same, current results partially confirm and are complementary to the ones from Neuenschwander et al. [4], who listed a few wild spices as priority for conservation in Benin. Actually, there are no single approaches to generate lists of species to include in conservation plans. In this study, we adopted a recent approach developed by Brehm et al. [21] and successfully implemented by Idohou et al. [47] in assessing priority crop wild relatives for conservation in Benin. This approach is very flexible and allows combination of several socio-economic and ecological criteria in different methods, in order to achieve the objectives of the study. It differs from the approach used by Teso et al. [80], Berlingeri and Crespo [81], and Khoury et al. [82] to identified priority crop wild relatives for conservation in Spain, Venezuela, and USA respectively. However, the approach is time consuming giving the number of criteria considered and the different methods it combines to overcome the potential subjectivity that may result from attribution of scores and weights to each criterion. Besides, its implementation requires a lot of information that are not often available for most wild species, particularly the ones that are neglected by government and researchers. This being true for the functional group of wild spices in Benin, this prioritization exercise should be updated when more new information will be available on the species. Moreover, results from this approach may not necessarily reflect the conservation significance of each priority species as perceived by local people. This could greatly affect the acceptability and the success of any conservation action of wild spices at local level, since the communities’ interests may have been overlooked [20]. Indeed, the criteria used here may not correspond to those used by communities in their evaluation of the value of wild spices and much less to their perception of threatened species.

## Conclusion

This is a pioneer study in the field of wild spices investigation across habitats in Benin. It highlights how geographic location, sociolinguistic groups, and gender influence the traditional knowledge and the subsequent use pattern of the wild spices. Indeed, people in Bassila phytodistrict, especially Tchabè, are more knowledgeable on the use of wild spices. Likewise, male informants value the wild spices much more than women. Domestication and improvement programs of these species should take into account this traditional knowledge. The most culturally important species and uses are all functions of sociolinguistic grouping and vary according to phytodistrict. Based on the local perception of the nutritional value of wild spices, the study revealed that people give them a high nutritional value, while at the same time using them mostly for medicinal purposes. In addition, the wild spices are perceived in decline by local populations, mainly due to high anthropogenic pressures and to some extent drought. Besides, the study provides insights into which species in this study area should be given more priority in an active conservation. For effective conservation and sustainable management of wild spices, scientists must provide substantial data on such aspects as morphological and genetic diversity within the species and breaking of seed dormancy. We proposed the integration of the wild spices into home gardens and agroforestry-based systems in the sociolinguistic areas of Tchabè, in the phytodistrict of Bassila. This will ensure the conservation of the resources while helping the diversification of household incomes and enhancing nutritional and food security.

## Abbreviations

AIC: Akaike information criteria
ANOSIM: Analysis of similarities
BIC: Bayesian information criteria
BRS: Binomial Ranking System
CA: Correspondence analysis
CI: Cultural importance index
CRS: Compound Ranking System
*F*_ic_: Informants consensus factor
GLM: Generalized linear model
IUCN: International Union for Conservation of Nature
NTFPs: Non-timber forest products
PROTA: Plant Resources of Tropical Africa
PSP: Point Scoring Procedure
PSPW: Point Scoring Procedure with Weighting
SGZ: Sudano-Guinean Zone
TK: Traditional knowledge
UR: Use report
UV: Use value
